# Supplementary material for: Histone H3K4me3 modification is a transgenerational epigenetic signal for lipid metabolism in Caenorhabditis elegans
Source: Nat Commun. 2022 Feb 9;13:768. doi: 10.1038/s41467-022-28469-4 (PMC8828817; doi:10.1038/s41467-022-28469-4)
Supplement: Supplementary file 1 — Supplementary information [file 41467_2022_28469_MOESM1_ESM.pdf]

Histone H3K4me3 modification is a transgenerational epigenetic signal for lipid metabolism in *Caenorhabditis elegans*

Qin-Li Wan [1, 2, 3, #], Xiao Meng [1, 2, #], Chongyang Wang [1, 2], Wenyu Dai [1, 2], Zhenhuan Luo [1, 2], Zhinan Yin [1,2], Zhenyu Ju [4], Xiaodie Fu [1, 2], Jing Yang [1, 2], Qunshan Ye [1, 2], Zhan-Hui Zhang [5], Qinghua Zhou [1, 2, *]

[1] The Sixth Affiliated Hospital of Jinan University, Jinan University, Dongguan, 523560, Guangdong, China

[2] The Biomedical Translational Research Institute, Faculty of Medical Science, Jinan University, Guangzhou, 510632, Guangdong, China

[3] Department of pathogen biology, school of medicine, Jinan University, Guangzhou 510632, China

[4] Key Laboratory of Regenerative Medicine of Ministry of Education, Institute of Aging and Regeneration Medicine, Jinan University, Guangzhou, Guangdong, 510632, China

[5] The First Affiliated Hospital, Jinan University, Guangzhou, Guangdong, 510632, China

[#] Qin-Li Wan and Xiao Meng have contributed equally to this work

*Corresponding author: Dr. Qinghua Zhou

The Sixth Affiliated Hospital of Jinan University, Jinan University, Dongguan,

523560, Guangdong, China

Phone: +86 20 85222787; Fax: +86 20 85222787

E-mail address: gene@email.jnu.edu.cn

Supplementary Information

Contents:

Supplementary Fig. 1 TEI of lipid accumulation induced by HFD.

Supplementary Fig. 2 The effect of HFD on lifespan of *C. elegans*.

Supplementary Fig. 3 Epistasis analyses of *nhr-49*, *nhr-80*, *sbp-1* and *daf-16* in multigenerational obesogenic effects induced by HFD.

Supplementary Fig. 4 NHR-49, NHR-80, SBP-1, FAT-6 and FAT-7 are responsible for TEI of lipid accumulation induced by HFD.

Supplementary Fig. 5 HFD-induced TEI of lipid accumulation requires DAF-16.

Supplementary Fig. 6 Other histone modifications including H3K27me3 and H3K36me3 are not required for transgenerational inheritance of lipid accumulation induced by the HFD.

Supplementary Fig. 7 Analyses of differentially expressed genes from P0 worms with or without HFD in wild-type worms.

Supplementary Fig. 8 DAF-16, NHR-80 and NHR-49 did not affect the elevation of H3K4me3 induced by HFD.

Supplementary Fig. 9 DAF-16 mediated TEI induced by HFD in a DAF-2 independent manner.

Supplemental Fig. 10 Tissue-specific RNAi of *sbp-1*.

Supplementary Table 1 The primers used in RT-qPCR and Chip-qPCR.

Supplementary Fig. 1

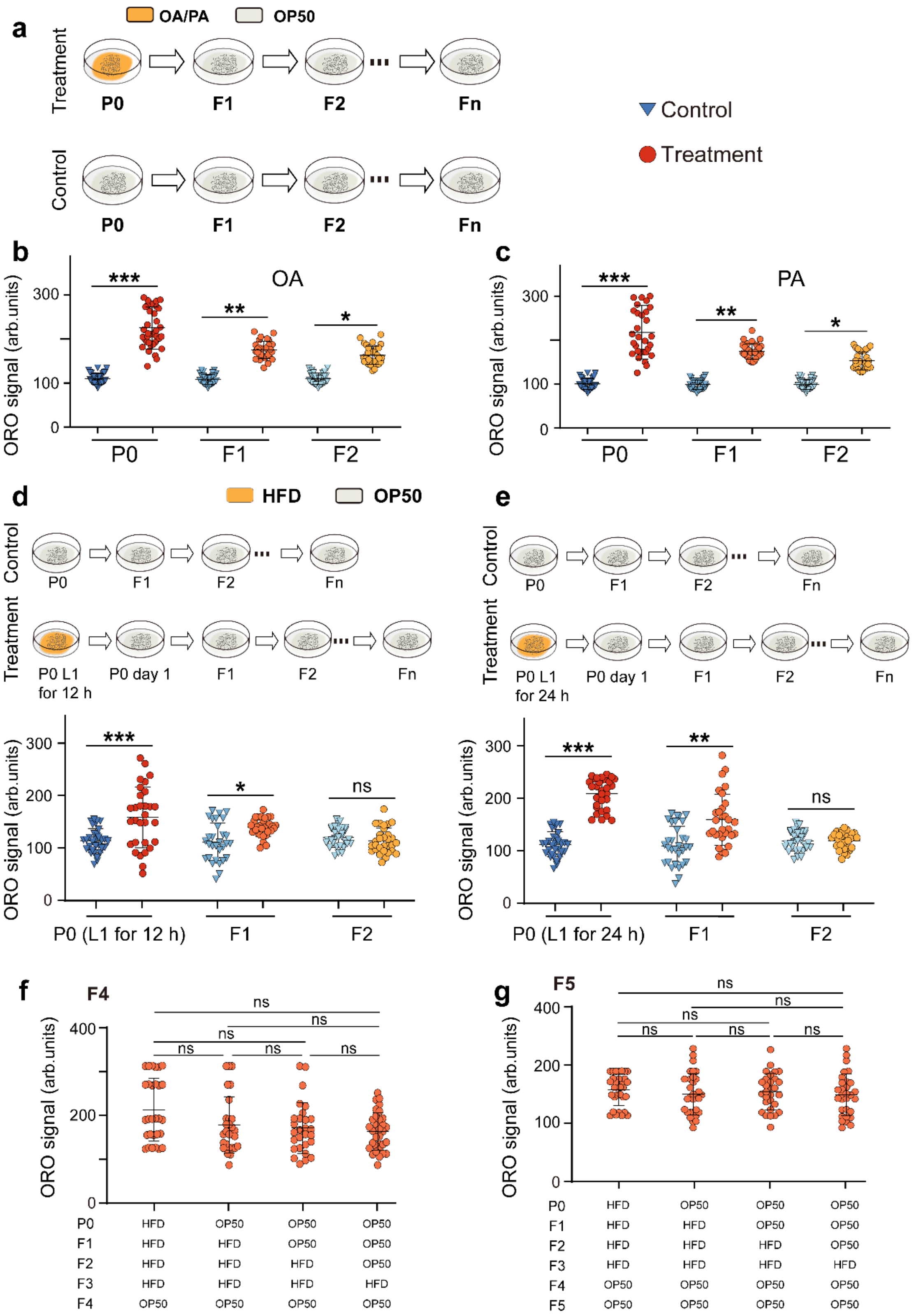

Supplementary Fig. 1 TEI of lipid accumulation induced by HFD. (a-c)

Transgenerational inheritance of lipid accumulation induced by OA (n ≥ 29 per condition) **(b)** or PA (n ≥ 29 per condition) **(c)** in wild-type *C. elegans*. PA: palmitoleic acid; OA: oleic acid. **(d, e)** Quantification of ORO staining of recovered F1 or F2 from parents fed with HFD from L1 larvae for 12 h (n ≥ 30 per condition) **(d)** or 24 h (n ≥ 30 per condition) **(e)**. For Fig. **b-e**, graph data are presented as mean ± SD, statistical analyses were performed by unpaired two-tailed Student's t-test; *** $p < 0.001$, ** $p < 0.01$, * $p < 0.05$, and ns, no significance. The ORO staining values of the replicated tests are listed in Source Data file. **(f, g)** Statistical analyses of ORO staining of treatment animals from Fig. 1f & g by ANOVA analysis (n ≥ 28 per condition; ns, no significance). Source data are provided as a Source Data file.

Supplementary Fig. 2

a

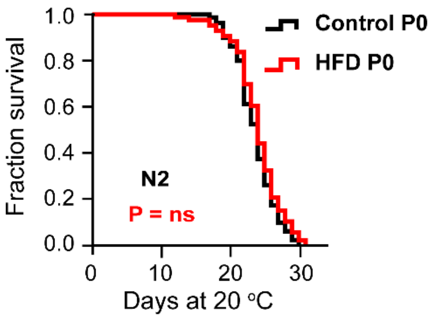

b

Strains	Treatments	Mean Lifespan ± SEM (days)	*P* value VS control	N
N2 (WT)				
EXP. 1	20 °C / control P0	22.176±0.428		68
EXP. 1	20 °C / HFD P0	22.338±0.420	P=0.748	74
EXP. 2	20 °C / control P0	22.106±0.389		66
EXP. 2	20 °C / HFD P0	22.254±0.402	P=0.648	71
EXP. 3	20 °C / control P0	21.788±0.278		110
EXP. 3	20 °C / HFD P0	22.142±0.206	P=0.031	113
EXP. 4	20 °C / control P0	22.723±0.247		112
EXP.4	20 °C / HFD P0	21.966±0.271	P=0.109	119

Supplementary Fig. 2 The effect of HFD on lifespan of *C. elegans*. (a) Survival analyses of wild-type N2 worms fed with HFD from L1 larvae to young adults. **(b)** Detailed lifespan values from repeat experiments. Lifespan was analyzed using the Kaplan-Meier test, *P* values were calculated using the log-rank test.

Supplementary Fig. 3

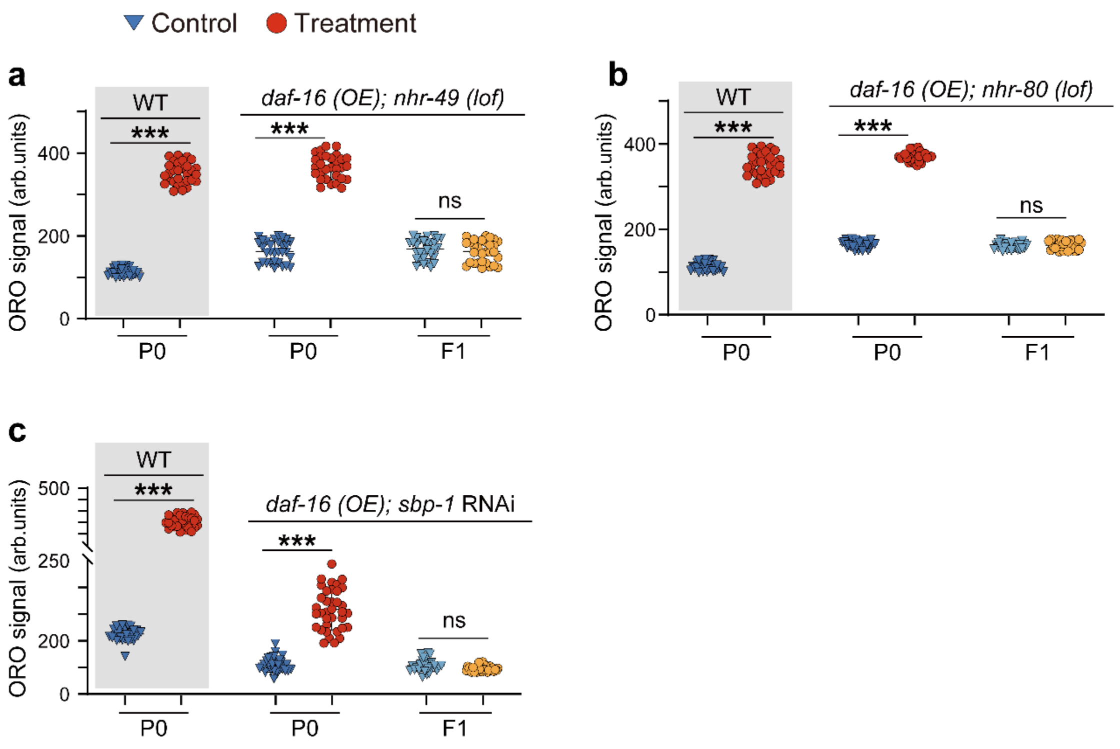

Supplementary Fig. 3 Epistasis analyses of *nhr-49*, *nhr-80*, *sbp-1* and *daf-16* in multigenerational obesogenic effects induced by HFD. Transgenerational inheritance test of HFD-induced lipid accumulation in *daf-16(OE); nhr-49(lof) double* mutant (n ≥ 30 per condition) **(a)**, *daf-16(OE); nhr-80(lof) double* mutant (n ≥ 30 per condition) **(b)**, and *daf-16(OE); sbp-1* RNAi mutant (n ≥ 30 per condition) **(c).** Graph data are presented as mean ± SD, statistical analyses were performed by unpaired two-tailed Student's t-test; *** $p < 0.001$, ns, no significance. Source data are provided as a Source Data file.

Supplementary Fig. 4

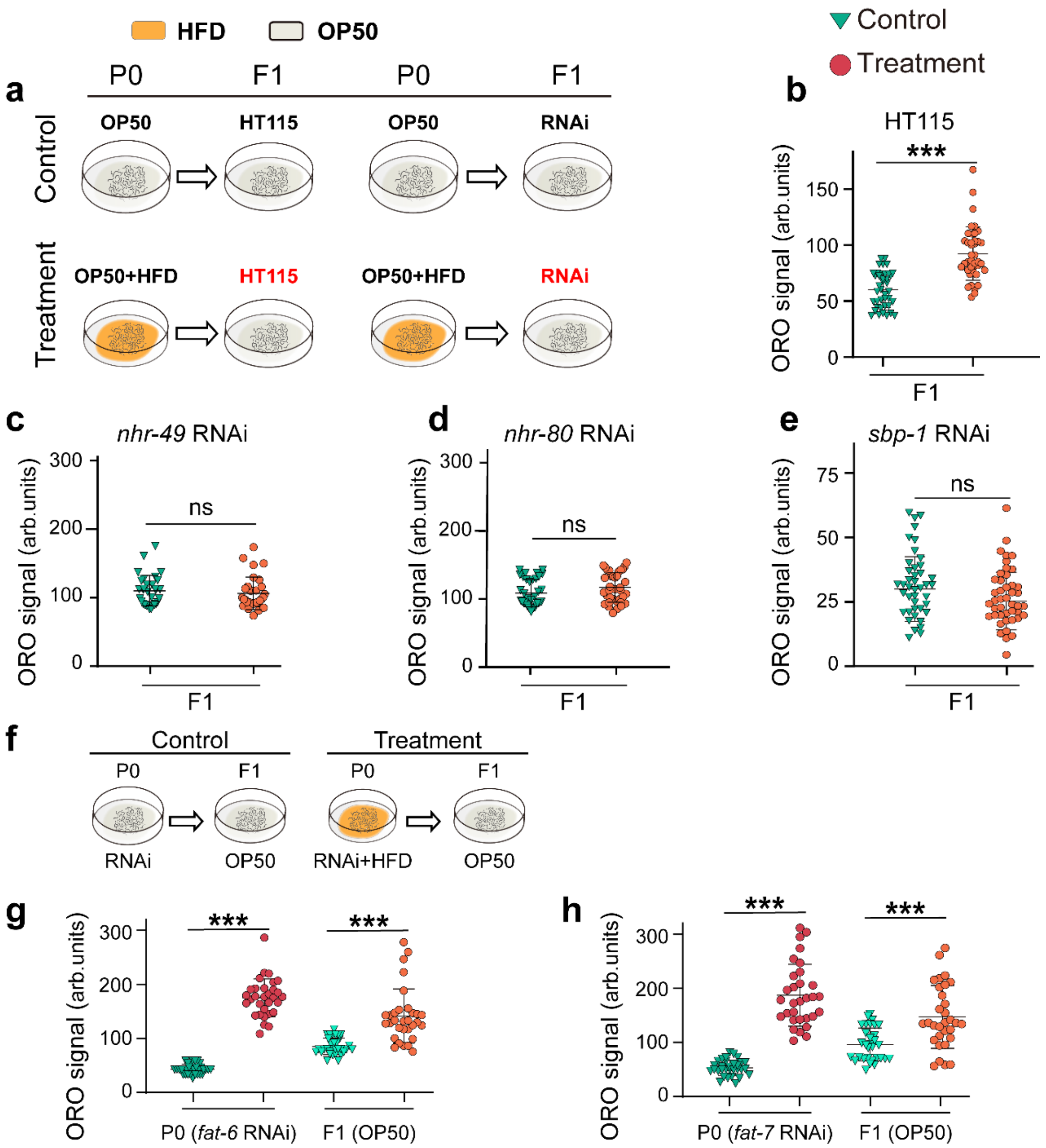

Supplementary Fig. 4 NHR-49, NHR-80, SBP-1, FAT-6 and FAT-7 are responsible for TEI of lipid accumulation induced by HFD. (a) Experimental scheme. **(b-e)** P0 worms were fed with or without HFD, and F1 progeny were exposed to control (HT115) ($n \geq 27$ per condition) **(b)**, *nhr-49* RNAi ($n \geq 31$ per condition) **(c)**, *nhr-80* RNAi ($n \geq 32$ per condition) **(d)** and *sbp-1* RNAi ($n \geq 36$ per condition) **(e)** to determine the

requirement of *nhr-49*, *nhr-80* and *sbp-1* in the F1 generation. **(f-h)** P0 animals were fed with or without HFD and subjected to *fat-6* RNAi (n ≥ 31 per condition) **(g)** or *fat-7* RNAi (n ≥ 31 per condition) **(h)**, and then lipid levels of F1 progeny fed with OP50 were analyzed. The ORO staining values of the replicated tests are listed in Source Data file. For Fig. **b-e** and **g-h,** graph data are presented as mean ± SD; Student's t-test; *** $p < 0.001$, ns, no significance). Source data are provided as a Source Data file.

Supplementary Fig. 5

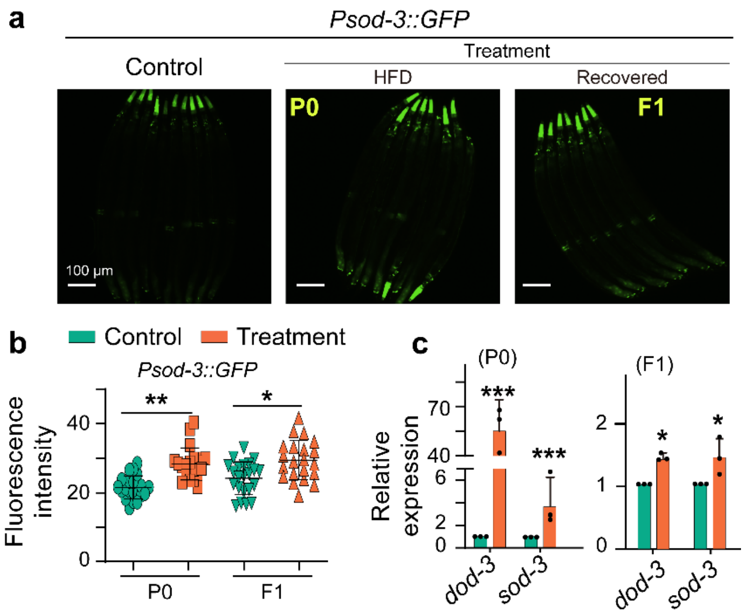

Supplementary Fig. 5 HFD-induced TEI of lipid accumulation requires DAF-16. **(a, b)** The SOD-3::GFP expression of worms fed with or without HFD and their recovered F1 progeny (mean ± SD; two-sided Student's t-test; n ≥ 21; ** $p < 0.01$ and * $p < 0.05$) (**a** is representative of three repeat experiments). **(c)** mRNA levels of target genes of DAF-16 (*sod-3* and *dod-3*) in P0 animals fed with or without the HFD and their recovered progeny (mean ± SD; unpaired two-tailed Student's t-test; n = 3 biologically independent samples; *** $p < 0.001$ and * $p < 0.05$).

Supplementary Fig. 6

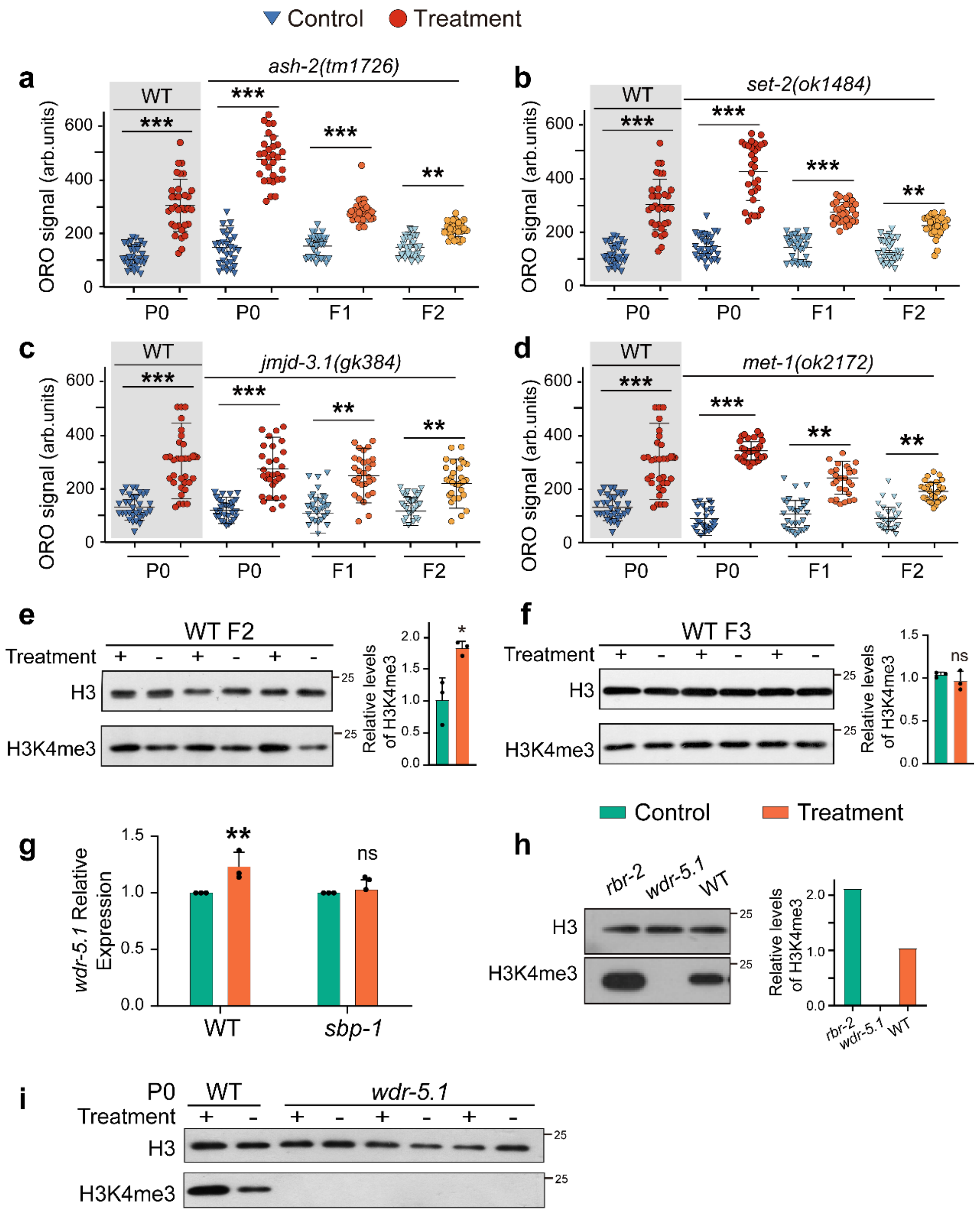

Supplementary Fig. 6 Other histone modifications including H3K27me3 and H3K36me3 are not required for transgenerational inheritance of lipid accumulation induced by the HFD. Transgenerational inheritance test of HFD-

induced lipid accumulation in *ash-2* mutant (n $\geq$ 30 per condition) **(a)**, *set-2* mutant (n $\geq$ 30 per condition) **(b)**, *jmjd-3.1* mutant (n $\geq$ 30 per condition) **(c)** and *met-1* mutant (n $\geq$ 29 per condition) **(d)**. For Fig. **a-d**, graph data are presented as mean ± SD, statistical analyses were performed by unpaired two-tailed Student's t-test; *** $p < 0.001$, and ** $p < 0.01$, and the ORO staining values of the replicated tests are listed in Source Data file. **(e, f)** Western blots (left panels) and quantification (right panels) of histone H3K4me3 modification of WT animals in F2 and F3 progeny from parents fed with HFD (means ± SD, unpaired two-tailed Student's t-test, n = 3 biologically independent samples, * $p < 0.05$, ns, no significance). H3 was shown as loading control. **(g)** *wdr-5.1* mRNA level in WT or *sbp-1* mutant fed with or without HFD. Mean ± SD; Student's t-test; n = 3 biologically independent samples; ** $p < 0.01$ and ns: no significance. **(h)** Western blots (left panels) and quantification (right panels) of histone H3K4me3 modification in *rbr-2* mutant, *wdr-5.1* mutant and WT N2 (n = 1). **(i)** The levels of histone H3K4me3 modification in *wdr-5.1* mutant fed with or without HFD (n = 3 biologically independent samples). Source data are provided as a Source Data file

Supplementary Fig. 7

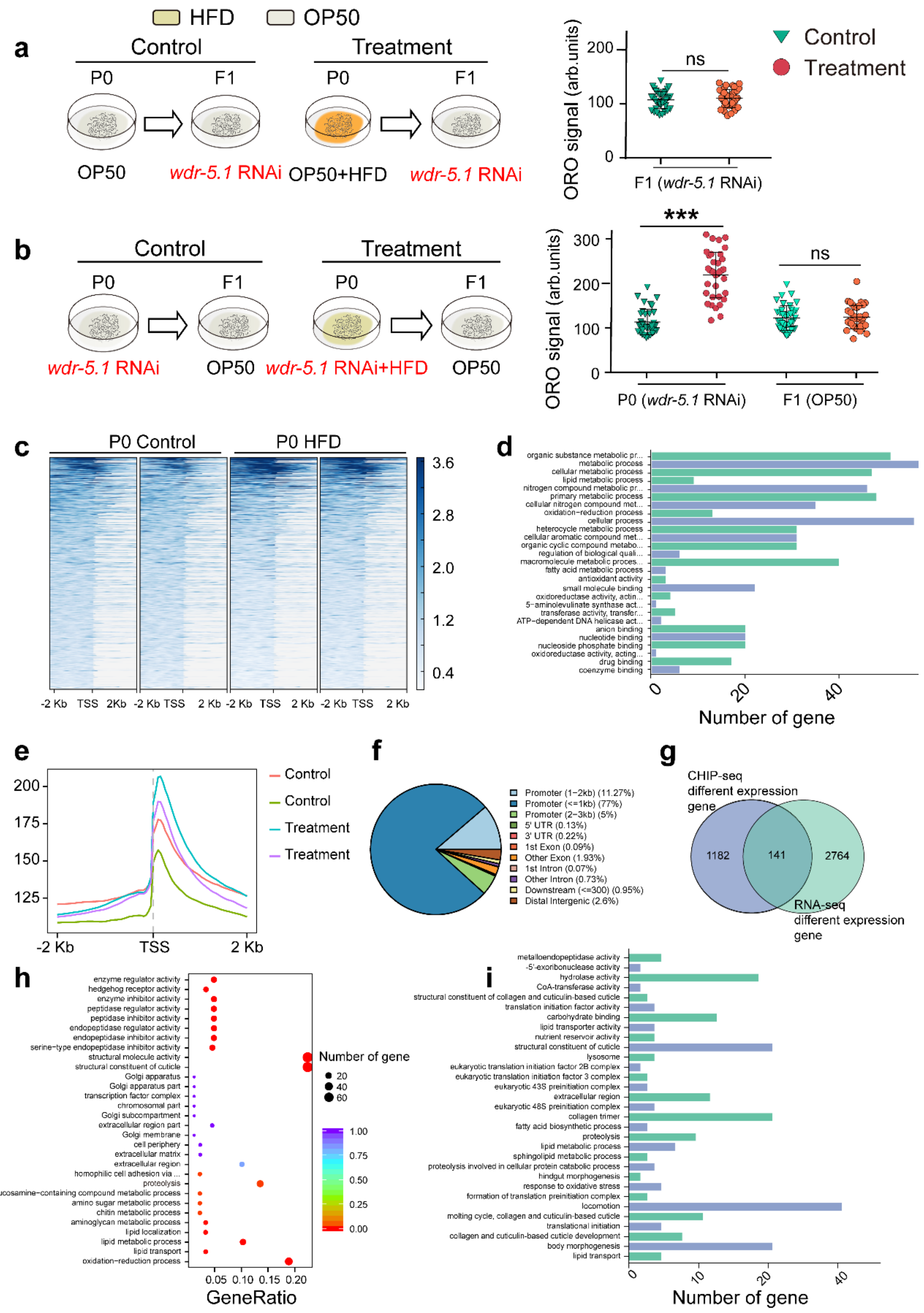

Supplementary Fig. 7 Analyses of differentially expressed genes from P0 worms with or without HFD in wild-type worms. (a) P0 worms were fed with or without

HFD, and F1 progeny were exposed to *wdr-5.1* RNAi to determine the requirement of *wdr-5.1* in the F1 generation (n ≥ 28 per condition). **(b)** P0 animals were fed with or without HFD and subjected to *wdr-5.1* RNAi, and then lipid level of F1 progeny was analyzed (n ≥ 31 per condition). Graph data are presented as mean ± SD, statistical analyses were performed by unpaired two-tailed Student's t-test; *** p <0.001, ns, no significance. Source data are provided as a Source Data file. **(c)** Heatmap of H3K4me3 ChIP signals approximately 2 kb upstream and downstream of regions that were differentially enriched in P0 generation between the treatment and control groups. The peaks were ranked in a descending order of H3K4me3 intensity within each cluster. The data shown refer to common genes as indicated by two independent assays. **(d)** Box plots of gene ontology term (GO) analysis of the differential H3K4me3 modification in the P0 animals fed with HFD or OP50. **(e)** Profile plot of H3K4me3 modification in the WT animals fed with HFD or OP50. **(f)** Pie chart of the genomic distribution of H3K4me3 differentially accumulated peaks for P0 animals fed with HFD or OP50. **(g)** Venn diagram comparing H3K4me3 differentially accumulated genes and the levels of mRNA dysregulated genes upon P0 animals fed with HFD. **(h)** Dot plots of gene ontology term (GO) analysis of the mRNAs differentially regulated in the P0 animals fed with HFD. **(i)** Box plots of gene ontology term (GO) analysis of the overlapping genes that were significantly changed in both CHIP-seq and RNA-seq data.

Supplementary Fig. 8

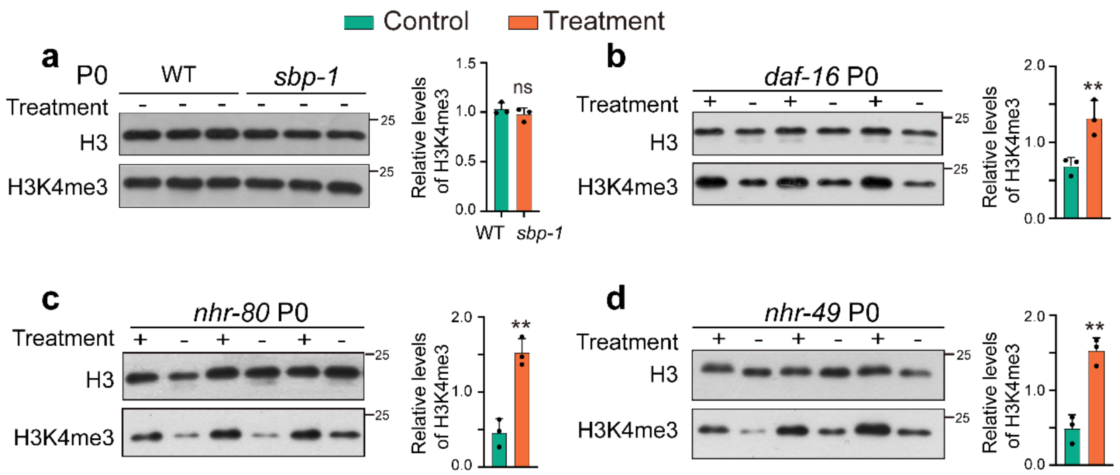

Supplementary Fig. 8 DAF-16, NHR-80 and NHR-49 did not affect the elevation of H3K4me3 induced by HFD. (a) Western blots (left panels) and quantification (right panels) of H3K4me3 modification level in WT or *sbp-1* mutant fed with normal food. **(b-d)** Western blots (left panels) and quantification (right panels) of H3K4me3 modification level in *daf-16* mutant **(b)**, *nhr-80* mutant **(c)** and *nhr-49* mutant **(d)**, when animals fed with or without the HFD. Means ± SD, unpaired two-tailed Student's t-test, n = 3 biologically independent samples, ** $p < 0.01$, ns, no significance. H3 was shown as loading control. Source data are provided as a Source Data file.

Supplementary Fig. 9

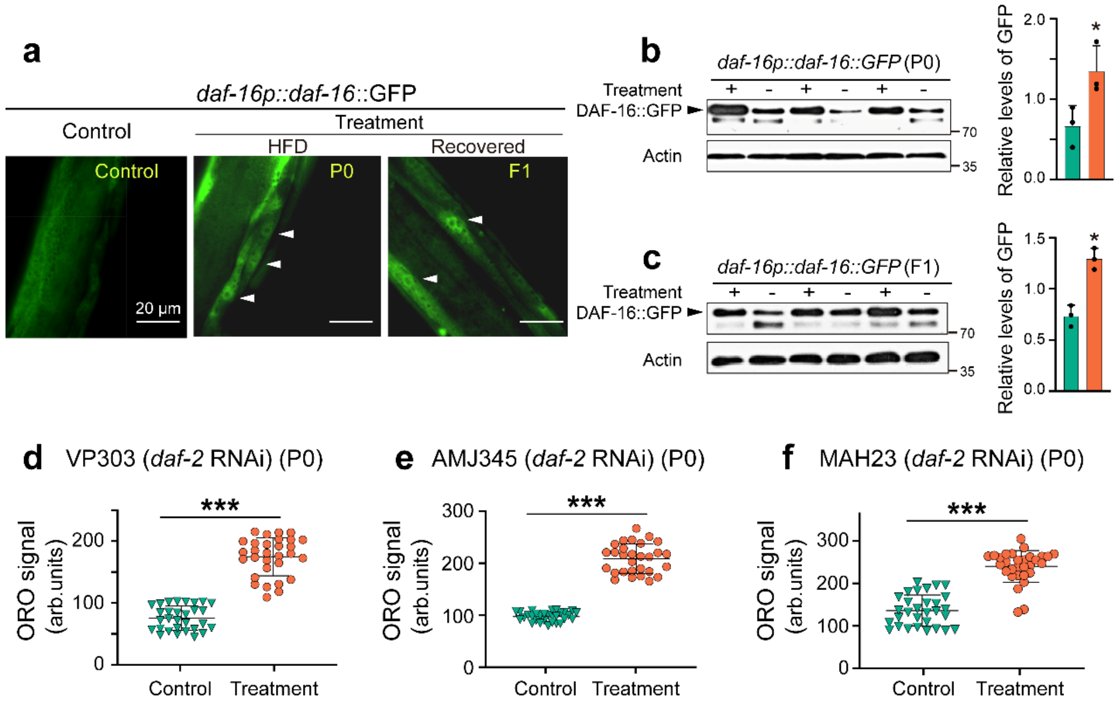

Supplementary Fig. 9 DAF-16 mediated TEI induced by HFD in a DAF-2 independent manner. Nuclear localization (representative of three experiments) **(a)** and wstern blots (left panels) and quantification (right panels) of DAF-16::GFP expression in the P0 transgenic worms fed with or without HFD (n = 3 biologically independent samples) **(b)** and their recovered F1 progeny (n = 3 biologically independent samples) **(c)** (means ± SD, unpaired two-tailed Student's t-test, * $p < 0.05$). Actin was shown as loading control. **(d-f)** Quantification of ORO staining in animals subjected to intestine-specific *daf-2* RNAi (n ≥ 30 per condition) **(d)**, germline and intestine-specific *daf-2* RNAi (n ≥ 30 per condition) **(e)**, and germline specific *daf-2* RNAi (n ≥ 30 per condition) **(f)**. Graph data are presented as mean ± SD, statistical analyses were performed by unpaired two-tailed Student's t-test; *** $p < 0.001$, and Source data are provided as a Source Data file.

Supplementary Fig. 10

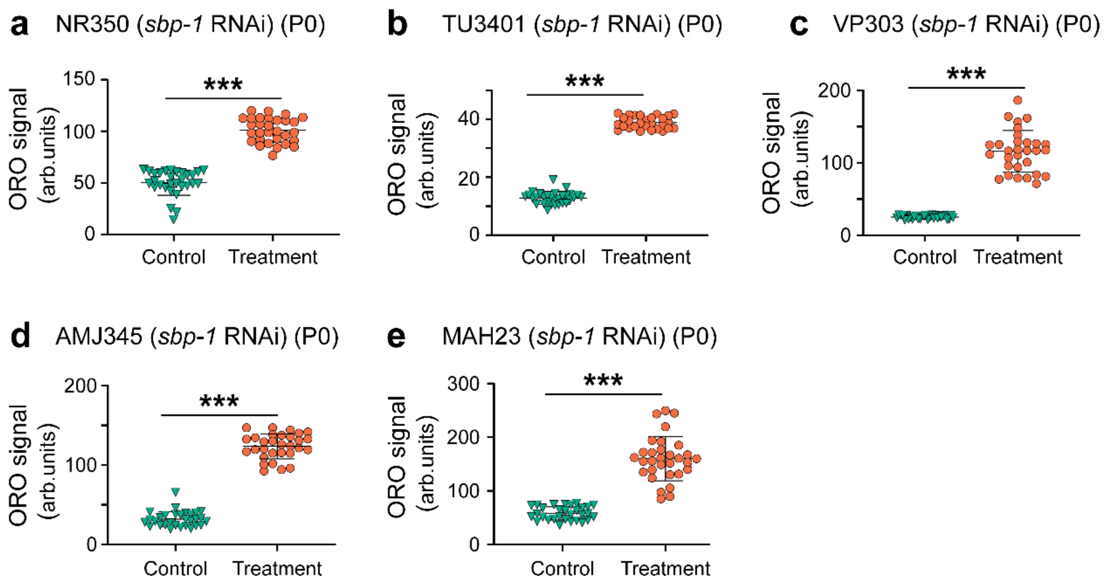

Supplementary Fig. 10 Tissue-specific RNAi of *sbp-1*. (a-e) Quantification of ORO staining in animals subjected to muscle-specific *sbp-1* RNAi (n ≥ 30 per condition) **(a)**, neuron-specific *sbp-1* RNAi (n ≥ 30 per condition) **(b)**, intestine-specific *sbp-1* RNAi (n ≥ 30 per condition) **(c)**, germline and intestine-specific *sbp-1* RNAi (n ≥ 30 per condition) **(d)**, germline specific *sbp-1* RNAi (n ≥ 30 per condition) **(e)**. Graph data are presented as mean ± SD, statistical analyses were performed by unpaired two-tailed Student's t-test; *** $p < 0.001$, and Source data are provided as a Source Data file.

Supplementary Table 1. The primers used in RT-qPCR and Chip-qPCR

Primer sequences used for RT-qPCR and Chip-qPCR (5’→3’):		
Primer name	Used for	Sequences(5'-3')
cdc-42 Forward	RT-qPCR	CTGCTGGACAGGAAGATTACG
cdc-42 Reversed	RT-qPCR	CTCGGACATTCTCGAATGAAG
dod-3 Forward	RT-qPCR	CGTATATGGACCCAGCTAATG
dod-3 Reverse	RT-qPCR	ATGAACACCGGCTCATTC
sod-3 Forward	RT-qPCR	AGCATCATGCCACCTACGTGA
sod-3 Reverse	RT-qPCR	CACCACCATTGAATTTCAGCG
daf-16 Forward	RT-qPCR	TCGTCGTCTCGTGTTTCTCCA
daf-16 Reverse	RT-qPCR	TTCCATAGGCACCCGGTAGTG
sbp-1 Forward	RT-qPCR	CATGAATTCATTCGAGGGAGACGTCCC
sbp-1 Reverse	RT-qPCR	CATGAATTCCTGATGTGGAGTCATCGC
nhr-49 Forward	RT-qPCR	GTCGTTATTGTCGCTTTCAA
nhr-49 Reverse	RT-qPCR	TCCGACACCGTTGCTGTTTC
nhr-80 Forward	RT-qPCR	TGAGGTTCAGGAGCCAAATAG
nhr-80 Reverse	RT-qPCR	GAAGGAGGTGGACGATGAGA
M199.2 Forward	RT-qPCR	ACCGCAGAGATTTGGGACAG
M199.2 Reverse	RT-qPCR	TTTGCGAAGTTGCGTAGCAC
gmd-2 Forward	RT-qPCR	GTGGTGAACTATCGGGAGGC
gmd-2 Reverse	RT-qPCR	AAGATTGCAGAACTCGCGGA

csn-2 Forward	RT-qPCR	TTACGAGTTGGCGACACAGG
csn-2 Reverse	RT-qPCR	CCAGGAAACGACCATCACGA
fat-5 Forward	RT-qPCR	GGGCTACAGTTGGATGGGTATT
fat-5 Reverse	RT-qPCR	CGGGTCAGCATCAGTATCCG
fat-6 Forward	RT-qPCR	AAGATTGAGAAGGACGGCGG
fat-6 Reverse	RT-qPCR	TCACGGTTTGCCATTTTGCC
fat-7 Forward	RT-qPCR	AAGGAGCATGGAGGCAAACT
fat-7 Reverse	RT-qPCR	TTCTCAACGGCGGAAACAGA
daf-16 Forward	ChIP-qPCR	TTGGAGAAAGAGAGCGAGCG
daf-16 Reverse	ChIP-qPCR	CGGGCACAGGAGAAGAAGAG
sbp-1 Forward	ChIP-qPCR	GTCGGATGCAAGTCCTCACTTA
sbp-1 Reverse	ChIP-qPCR	GTGAGATGTCGTGGGCATATTG
nhr-49 Forward	ChIP-qPCR	TGTCCTTCTAACCGACTGGC
nhr-49 Reverse	ChIP-qPCR	CAAAGCTAATTGGGACCGGC
nhr-80 Forward	ChIP-qPCR	ACACCACACTCTCTCGCCT
nhr-80 Reverse	ChIP-qPCR	TCCCGCCAAACTGTAGAACATA
M199.2 Forward	ChIP-qPCR	TTATACACAAGAGACGACGGC
M199.2 Reverse	ChIP-qPCR	TGACGCAGTGAATTGTTCGC
gmd-2 Forward	ChIP-qPCR	CACTCGAAGCGATATGGAAGGA
gmd-2 Reverse	ChIP-qPCR	TGAATAGTTCCCGGATACGCAG
csn-2 Forward	ChIP-qPCR	TGGACAGAATAATGGGTGACGA
csn-2 Reverse	ChIP-qPCR	ACCGAACTTTTCCTGGGTCTC

fat-5 Forward	ChIP-qPCR	AGACCTGACCTGATCGAGCTTT
fat-5 Reverse	ChIP-qPCR	TAACTACTGCAAAAGAAGGGGCG
fat-6 Forward	ChIP-qPCR	GCTTGCCCAGACATACCACA
fat-6 Reverse	ChIP-qPCR	GGTGATTGGTGTTTGCACCG
fat-7 Forward	ChIP-qPCR	CGCTCGCCCCATTGAAATAC
fat-7 Reverse	ChIP-qPCR	AATCCTCGCTTCTGGCTTGG
