# Supplementary material for: Histone H3K4me3 modification is a transgenerational epigenetic signal for lipid metabolism in Caenorhabditis elegans
Source: Nat Commun. 2022 Feb 9;13:768. doi: 10.1038/s41467-022-28469-4 (PMC8828817; doi:10.1038/s41467-022-28469-4)
Supplement: Supplementary file 3 — Reporting Summary [file 41467_2022_28469_MOESM3_ESM.pdf]

nature research

Corresponding author(s): Qinghua Zhou

Last updated by author(s): Oct 13, 2021

Reporting Summary

Nature Research wishes to improve the reproducibility of the work that we publish. This form provides structure for consistency and transparency in reporting. For further information on Nature Research policies, see our Editorial Policies and the Editorial Policy Checklist.

Statistics

For all statistical analyses, confirm that the following items are present in the figure legend, table legend, main text, or Methods section.

n/a	Confirmed	
[ ]	[x]	The exact sample size (*n*) for each experimental group/condition, given as a discrete number and unit of measurement
[ ]	[x]	A statement on whether measurements were taken from distinct samples or whether the same sample was measured repeatedly
[ ]	[x]	The statistical test(s) used AND whether they are one- or two-sided<br>*Only common tests should be described solely by name; describe more complex techniques in the Methods section.*
[x]	[ ]	A description of all covariates tested
[x]	[ ]	A description of any assumptions or corrections, such as tests of normality and adjustment for multiple comparisons
[ ]	[x]	A full description of the statistical parameters including central tendency (e.g. means) or other basic estimates (e.g. regression coefficient) AND variation (e.g. standard deviation) or associated estimates of uncertainty (e.g. confidence intervals)
[x]	[ ]	For null hypothesis testing, the test statistic (e.g. *F*, *t*, *r*) with confidence intervals, effect sizes, degrees of freedom and *P* value noted<br>*Give P values as exact values whenever suitable.*
[x]	[ ]	For Bayesian analysis, information on the choice of priors and Markov chain Monte Carlo settings
[x]	[ ]	For hierarchical and complex designs, identification of the appropriate level for tests and full reporting of outcomes
[x]	[ ]	Estimates of effect sizes (e.g. Cohen's *d*, Pearson's *r*), indicating how they were calculated

Our web collection on statistics for biologists contains articles on many of the points above.

Software and code

Policy information about availability of computer code

Data collection	The commercial software used for data collection are specified in the methods section.
Data analysis	SPSS; Graphpad Prism; Image J; Excel; DAVID Bioinformatics Resources.

For manuscripts utilizing custom algorithms or software that are central to the research but not yet described in published literature, software must be made available to editors and reviewers. We strongly encourage code deposition in a community repository (e.g. GitHub). See the Nature Research guidelines for submitting code & software for further information.

Data

Policy information about availability of data

All manuscripts must include a data availability statement. This statement should provide the following information, where applicable:

- Accession codes, unique identifiers, or web links for publicly available datasets
- A list of figures that have associated raw data
- A description of any restrictions on data availability

All data generated or analyzed during this study are included in this manuscript and the supplementary files.

Field-specific reporting

Life sciences study design

All studies must disclose on these points even when the disclosure is negative.

Sample size	Sample sizes each experiment were chosen in accordance with the general standards and prior publications in the respective fields.
Data exclusions	There are no data exclusions.
Replication	All attempts at replication were successful.
Randomization	This is not relevant to our study, because worms of each genotype were chosen randomly and used for analyses.
Blinding	During data collection, researchers were blinded to group allocation.

Reporting for specific materials, systems and methods

We require information from authors about some types of materials, experimental systems and methods used in many studies. Here, indicate whether each material, system or method listed is relevant to your study. If you are not sure if a list item applies to your research, read the appropriate section before selecting a response.

Materials & experimental systems

n/a	Involved in the study	
☐	☒	Antibodies
☒	☐	Eukaryotic cell lines
☒	☐	Palaeontology and archaeology
☐	☒	Animals and other organisms
☒	☐	Human research participants
☒	☐	Clinical data
☒	☐	Dual use research of concern

Methods

n/a	Involved in the study	
☐	☒	ChIP-seq
☒	☐	Flow cytometry
☒	☐	MRI-based neuroimaging

Antibodies

Antibodies used	H3 (Cell Signaling Technolog, H9715); H3K4me3 (Millipore, 04-745); H3K4me3 (Abcam ab8580)
Validation	All the validations and citations can be found on the manufacturer's websites.

Animals and other organisms

Policy information about studies involving animals; ARRIVE guidelines recommended for reporting animal research

Laboratory animals	The nematode Caenorhabditis elegans was used. All the strains used are described in methods.
Wild animals	No wild animals were used in this study.
Field-collected samples	This study did not involve samples collected from the field.
Ethics oversight	In china, no ethical approval is needed for experiments using C. elegans.

Note that full information on the approval of the study protocol must also be provided in the manuscript.

ChIP-seq

Data deposition

☒ Confirm that both raw and final processed data have been deposited in a public database such as GEO.

☒ Confirm that you have deposited or provided access to graph files (e.g. BED files) for the called peaks.

Data access links<br>*May remain private before publication.*	The high-through sequencing data generated and analyzed during this study are available from NCBI at the following accession code: PRJNA77044
Files in database submission	We provide FASTQ files

Genome browser session (e.g. UCSC)	FASTQ files can be convert to BigWig files, then BigWig files were visualized by Integrative Genomics Viewer (IGV).

Methodology

Replicates	Chip-seq data with two independent biological experiments.
Sequencing depth	All sequencing was paired-ended. Each sample was sequenced to a depth of about 20 Million mapped reads.
Antibodies	H3K4me3 (Abcam ab8580)
Peak calling parameters	regions of IP enrichment over background were identified by the MACS2 (version 2.1.0) peak calling software (q-value threshold of 0.05 was used for all data sets).
Data quality	q-value threshold of 0.05 was used for all data sets
Software	FastP, MACS2, ChIPseeker, DeepTools, R package and KOBAS software
