# Supplementary material for: Histone H3K4me3 modification is a transgenerational epigenetic signal for lipid metabolism in Caenorhabditis elegans
Source: Nat Commun. 2022 Feb 9;13:768. doi: 10.1038/s41467-022-28469-4 (PMC8828817; doi:10.1038/s41467-022-28469-4)
Supplement: Supplementary file 4 — Source Data [file 41467_2022_28469_MOESM4_ESM.zip › source data/Summary of C. elegans the ORO staining values-V2.docx]

**Summary of *C. elegans* the ORO staining values**

| Figure | Strains | Treatments | Mean ± SD  (arb. units) | *P* value  VS  control | N |
| --- | --- | --- | --- | --- | --- |
|  | **N2** |  |  |  |  |
| Fig. 1a-d | EXP.1 | 20 ℃/Control P0 | 106.42±34.65 |  | 33 |
|  | EXP.1 | 20 ℃/HFD P0 | 328.68±106.42 | <0.0001 | 33 |
|  | EXP.1 | 20 ℃/Control F1 | 113.18±24.57 |  | 29 |
|  | EXP.1 | 20 ℃/HFD F1 | 215.35±72.00 | <0.0001 | 29 |
|  | EXP.1 | 20 ℃/Control F2 | 118.34±19.49 |  | 31 |
|  | EXP.1 | 20 ℃/HFD F2 | 167.10±19.19 | 0.0047 | 31 |
|  |  |  |  |  |  |
|  | EXP.2 | 20 ℃/Control P0 | 104.63±33.16 |  | 28 |
|  | EXP.2 | 20 ℃/HFD P0 | 325.06±102.20 | <0.0001 | 28 |
|  | EXP.2 | 20 ℃/Control F1 | 105.50±23.65 |  | 33 |
|  | EXP.2 | 20 ℃/HFD F1 | 164.46±41.06 | <0.0001 | 33 |
|  | EXP.2 | 20 ℃/Control F2 | 115.43±37.01 |  | 30 |
|  | EXP.2 | 20 ℃/HFD F2 | 150.59±41.97 | 0.0038 | 31 |
|  |  |  |  |  |  |
|  | EXP.3 | 20 ℃/Control P0 | 133.01±38.03 |  | 28 |
|  | EXP.3 | 20 ℃/HFD P0 | 257.57±99.75 | <0.0001 | 28 |
|  | EXP.3 | 20 ℃/Control F1 | 105.87±28.54 |  | 31 |
|  | EXP.3 | 20 ℃/HFD F1 | 162.36±40.41 | <0.0001 | 31 |
|  | EXP.3 | 20 ℃/Control F2 | 109.30±31.97 |  | 29 |
|  | EXP.3 | 20 ℃/HFD F2 | 158.80±38.83 | 0.0042 | 33 |
|  |  |  |  |  |  |
|  | EXP.1 | 20 ℃/Control P0 | 131.93±30.74 |  | 29 |
|  | EXP.1 | 20 ℃/Egg yolk P0 | 317.59±56.51 | <0.0001 | 29 |
|  | EXP.1 | 20 ℃/OA P0 | 223.51±67.57 | <0.0001 | 29 |
|  | EXP.1 | 20 ℃/PA P0 | 221.05±64.95 | <0.0001 | 29 |
|  | EXP.1 | 20 ℃/OA+PA P0 | 239.16±66.06 | <0.0001 | 29 |
|  | EXP.1 | 20 ℃/OA+PA  +cholesterol P0 | 264.96±55.45 | <0.0001 | 29 |
|  |  |  |  |  |  |
|  | EXP.2 | 20 ℃/Control P0 | 111.12±27.03 |  | 29 |
|  | EXP.2 | 20 ℃/OA P0 | 234.41±82.42 | <0.0001 | 29 |
|  | EXP.2 | 20 ℃/PA P0 | 238.49±83.81 | <0.0001 | 29 |
|  | EXP.2 | 20 ℃/OA+PA P0 | 230.92±77.02 | <0.0001 | 29 |
|  | EXP.2 | 20 ℃/OA+PA  +cholesterol P0 | 288.81±75.73 | <0.0001 | 29 |
|  |  |  |  |  |  |
|  |  |  |  |  |  |
|  | **N2 F3 fed with HFD→F4，F5** | | |  |  |
| Fig. 1f, g | EXP.1 | 20 ℃/Control F4 | 105.99±23.39 |  | 33 |
|  | EXP.1 | 20 ℃/HFD F4 | 163.56±42.69 | <0.0001 | 33 |
|  | EXP.1 | 20 ℃/Control F5 | 116.47±34.81 |  | 31 |
|  | EXP.1 | 20 ℃/HFD F5 | 151.48±40.82 | 0.038 | 30 |
|  |  |  |  |  |  |
|  | EXP.2 | 20 ℃/Control F4 | 105.50±27.507 |  | 31 |
|  | EXP.2 | 20 ℃/HFD F4 | 162.65±39.37 | <0.0001 | 31 |
|  | EXP.2 | 20 ℃/Control F5 | 108.53±33.78 |  | 32 |
|  | EXP.2 | 20 ℃/HFD F5 | 159.58±39.32 | 0.0002 | 29 |
|  |  |  |  |  |  |
|  | **N2 F2 & F3 fed with HFD→F4，F5** | |  |  |  |
| Fig. 1f, g | EXP.1 | 20 ℃/Control F4 | 109.99±28.00 |  | 28 |
|  | EXP.1 | 20 ℃/HFD F4 | 171.55±57.99 | <0.0001 | 28 |
|  | EXP.1 | 20 ℃/Control F5 | 112.91±24.63 |  | 32 |
|  | EXP.1 | 20 ℃/HFD F5 | 157.80±35.57 | 0.0019 | 29 |
|  |  |  |  |  |  |
|  | EXP.2 | 20 ℃/Control F4 | 117.22±27.48 |  | 30 |
|  | EXP.2 | 20 ℃/HFD F4 | 179.90±56.53 | <0.0001 | 30 |
|  | EXP.2 | 20 ℃/Control F5 | 108.03±24.38 |  | 28 |
|  | EXP.2 | 20 ℃/HFD F5 | 153.40±30.40 | 0.003 | 28 |
|  |  |  |  |  |  |
|  | **N2 F1, F2 & F3 fed with HFD→F4，F5** | | |  |  |
| Fig. 1f, g | EXP.1 | 20 ℃/Control F4 | 120.26±30.67 |  | 28 |
|  | EXP.1 | 20 ℃/HFD F4 | 178.55±64.25 | <0.0001 | 28 |
|  | EXP.1 | 20 ℃/Control F5 | 105.67±22.43 |  | 29 |
|  | EXP.1 | 20 ℃/HFD F5 | 152.62±40.44 | 0.0037 | 31 |
|  |  |  |  |  |  |
|  | EXP.2 | 20 ℃/Control F4 | 103.15±26.47 |  | 28 |
|  | EXP.2 | 20 ℃/HFD F4 | 187.41±64.58 | <0.0001 | 28 |
|  | EXP.2 | 20 ℃/Control F5 | 118.73±29.72 |  | 28 |
|  | EXP.2 | 20 ℃/HFD F5 | 154.06±28.09 | 0.0018 | 28 |
|  |  |  |  |  |  |
|  | EXP.3 | 20 ℃/Control F4 | 108.71±23.43 |  | 33 |
|  | EXP.3 | 20 ℃/HFD F4 | 187.70±60.98 | <0.0001 | 33 |
|  | EXP.3 | 20 ℃/Control F5 | 104.28±25.83 |  | 29 |
|  | EXP.3 | 20 ℃/HFD F5 | 159.40±33.62 | 0.0017 | 28 |
|  |  |  |  |  |  |
|  | **N2 P0, F1, F2 & F3 fed with HFD→F4，F5** | | | |  |
| Fig. 1f, g | EXP.1 | 20 ℃/Control F4 | 114.43±25.04 |  | 29 |
|  | EXP.1 | 20 ℃/HFD F4 | 213.28±71.57 | <0.0001 | 29 |
|  | EXP.1 | 20 ℃/Control F5 | 118.34±19.37 |  | 31 |
|  | EXP.1 | 20 ℃/HFD F5 | 154.04±29.38 | 0.026 | 31 |
|  |  |  |  |  |  |
|  | EXP.2 | 20 ℃/Control F4 | 114.34±28.42 |  | 29 |
|  | EXP.2 | 20 ℃/HFD F4 | 192.14±71.13 | <0.0001 | 29 |
|  | EXP.2 | 20 ℃/Control F5 | 105.53±22.04 |  | 30 |
|  | EXP.2 | 20 ℃/HFD F5 | 157.83±27.69 | 0.0035 | 29 |
|  |  |  |  |  |  |
|  | EXP.3 | 20 ℃/Control F4 | 100.48±26.79 |  | 31 |
|  | EXP.3 | 20 ℃/HFD F4 | 182.03±62.64 | <0.0001 | 30 |
|  | EXP.3 | 20 ℃/Control F5 | 106.04±26.38 |  | 30 |
|  | EXP.3 | 20 ℃/HFD F5 | 155.78±45.56 | 0.002 | 29 |
|  |  |  |  |  |  |
|  | **N2 P1 (L1-L4 (HFD) →day1 (OP50)) → F1, F2 (OP50)** | | |  |  |
| Fig. 1e | EXP.1 | 20 ℃/Control P0 | 103.85±26.32 |  | 34 |
|  | EXP.1 | 20 ℃/HFD P0 | 212.07±55.42 | <0.0001 | 34 |
|  | EXP.1 | 20 ℃/Control F1 | 101.28±26.12 |  | 35 |
|  | EXP.1 | 20 ℃/HFD F1 | 164.91±46.84 | <0.0001 | 34 |
|  | EXP.1 | 20 ℃/Control F2 | 102.26±25.66 |  | 34 |
|  | EXP.1 | 20 ℃/HFD F2 | 144.52±32.56 | <0.0001 | 34 |
|  |  |  |  |  |  |
|  | EXP.2 | 20 ℃/Control P0 | 101.92±27.20 |  | 37 |
|  | EXP.2 | 20 ℃/HFD P0 | 207.99±51.36 | <0.0001 | 37 |
|  | EXP.2 | 20 ℃/Control F1 | 108.99±25.12 |  | 37 |
|  | EXP.2 | 20 ℃/HFD F1 | 166.99±39.62 | <0.0001 | 37 |
|  | EXP.2 | 20 ℃/Control F2 | 115.76±32.00 |  | 37 |
|  | EXP.2 | 20 ℃/HFD F2 | 147.21±31.71 | 0.0015 | 37 |
|  |  |  |  |  |  |
|  | EXP.3 | 20 ℃/Control P0 | 106.10±25.76 |  | 35 |
|  | EXP.3 | 20 ℃/HFD P0 | 212.58±54.24 | <0.0001 | 35 |
|  | EXP.3 | 20 ℃/Control F1 | 104.56±26.81 |  | 35 |
|  | EXP.3 | 20 ℃/HFD F1 | 172.75±43.11 | <0.0001 | 35 |
|  | EXP.3 | 20 ℃/Control F2 | 112.29±38.47 |  | 35 |
|  | EXP.3 | 20 ℃/HFD F2 | 152.18±41.73 | 0.002 | 35 |
|  |  |  |  |  |  |
|  | **N2 P1 (L1 for 12 hr or 24 hr (HFD) →day1 (OP50)) → F1, F2 (OP50)** | | | |  |
| Supplementary Fig. 1d&e | EXP.1 | 20 ℃ Control P0 | 110.89±21.56 |  | 30 |
|  | EXP.1 | 20 ℃HFD P0 12hr | 151.59±53.12 | 0.0125 | 30 |
|  | EXP.1 | 20 ℃ HFD P0 24hr | 199.88±26.07 | <0.0001 | 30 |
|  | EXP.1 | 20 ℃ Control F1 | 108.49±33.11 |  | 30 |
|  | EXP.1 | 20 ℃ HFD F1 12hr | 134.14±15.73 | 0.0189 | 30 |
|  | EXP.1 | 20 ℃ HFD F1 24hr | 153.00±43.51 | 0.0023 | 30 |
|  | EXP.1 | 20 ℃ Control F2 | 116.30±16.98 |  | 30 |
|  | EXP.1 | 20 ℃ HFD F2 12hr | 110.69±21.83 | 0.2704 | 30 |
|  | EXP.1 | 20 ℃ HFD F2 24hr | 117.03±14.16 | 0.8573 | 30 |
|  |  |  |  |  |  |
|  | EXP.2 | 20 ℃ Control P0 | 116.05±21.40 |  | 30 |
|  | EXP.2 | 20 ℃ FD P0 12hr | 157.57±21.96 | <0.0001 | 30 |
|  | EXP.2 | 20 ℃ HFD P0 24hr | 193.55±26.08 | <0.0001 | 30 |
|  | EXP.2 | 20 ℃ Control F1 | 105.08±18.99 |  | 30 |
|  | EXP.2 | 20 ℃ HFD F1 12hr | 131.03±31.10 | 0.0151 | 30 |
|  | EXP.2 | 20 ℃ HFD F1 24hr | 150.14±8.41 | <0.0001 | 30 |
|  | EXP.2 | 20 ℃ Control F2 | 115.30±15.01 |  | 31 |
|  | EXP.2 | 20 ℃ HFD F2 12hr | 116.45±12.41 | 0.7466 | 30 |
|  | EXP.2 | 20 ℃ HFD F2 24hr | 117.39±13.04 | 0.5642 | 30 |
|  |  |  |  |  |  |
|  | EXP.3 | 20 ℃ Control P0 | 118.77±15.58 |  | 31 |
|  | EXP.3 | 20 ℃ FD P0 12hr | 150.58±19.07 | <0.0001 | 30 |
|  | EXP.3 | 20 ℃ HFD P0 24hr | 188.56±36.74 | <0.0001 | 30 |
|  | EXP.3 | 20 ℃ Control F1 | 105.78±17.84 |  | 30 |
|  | EXP.3 | 20 ℃ HFD F1 12hr | 120.04±11.02 | 0.0266 | 30 |
|  | EXP.3 | 20 ℃ HFD F1 24hr | 143.44±7.12 | <0.0001 | 30 |
|  | EXP.3 | 20 ℃ Control F2 | 110.87±13.84 |  | 30 |
|  | EXP.3 | 20 ℃ HFD F2 12hr | 113.76±16.04 | 0.4583 | 30 |
|  | EXP.3 | 20 ℃ HFD F2 24hr | 116.62±16.79 | 0.1529 | 30 |
|  |  |  |  |  |  |
|  | **N2 (WT) fed with HFD male × control female** | | |  |  |
| Fig. 1i | EXP.1 | 20 ℃/Control F1 | 107.50±14.25 |  | 31 |
|  | EXP.1 | 20 ℃/HFD F1 | 151.80±16.75 | <0.0001 | 30 |
|  | EXP.2 | 20 ℃/Control F1 | 104.19±9.24 |  | 31 |
|  | EXP.2 | 20 ℃/HFD F1 | 153.83±20.56 | <0.0001 | 30 |
|  | EXP.3 | 20 ℃/Control F1 | 110.29±10.30 |  | 30 |
|  | EXP.3 | 20 ℃/HFD F1 | 150.44±14.62 | <0.0001 | 30 |
|  |  |  |  |  |  |
|  | **N2 (WT) fed with HFD female × control male** | | |  |  |
|  | EXP.1 | 20 ℃/Control F1 | 107.50±14.25 |  | 31 |
|  | EXP.1 | 20 ℃/HFD F1 | 176.94±61.33 | <0.0001 | 32 |
|  | EXP.2 | 20 ℃/Control F1 | 104.19±9.24 |  | 31 |
|  | EXP.2 | 20 ℃/HFD F1 | 166.67±50.89 | <0.0001 | 33 |
|  | EXP.3 | 20 ℃/Control F1 | 110.29±10.30 |  | 30 |
|  | EXP.3 | 20 ℃/HFD F1 | 189.69±66.33 | <0.0001 | 30 |
|  |  |  |  |  |  |
|  | **N2 without synchronization** | |  |  |  |
| Fig. 1h | EXP.1 | 20 ℃/Control P0 | 110.03±31.68 |  | 30 |
|  | EXP.1 | 20 ℃/HFD P0 | 356.21±46.44 | <0.0001 | 31 |
|  | EXP.1 | 20 ℃/Control F1 | 114.06±46.49 |  | 30 |
|  | EXP.1 | 20 ℃/HFD F1 | 215.19±18.73 | <0.0001 | 31 |
|  | EXP.1 | 20 ℃/Control F2 | 115.05±34.01 |  | 30 |
|  | EXP.1 | 20 ℃/HFD F2 | 155.32±22.75 | 0.0001 | 31 |
|  |  |  |  |  |  |
|  | EXP.2 | 20 ℃/Control P0 | 105.11±30.55 |  | 31 |
|  | EXP.2 | 20 ℃/HFD P0 | 367.34±48.11 | <0.0001 | 31 |
|  | EXP.2 | 20 ℃/Control F1 | 96.33±28.25 |  | 30 |
|  | EXP.2 | 20 ℃/HFD F1 | 214.16±21.09 | <0.0001 | 30 |
|  | EXP.2 | 20 ℃/Control F2 | 125.80±27.50 |  | 30 |
|  | EXP.2 | 20 ℃/HFD F2 | 169.96±26.74 | <0.0001 | 31 |
|  |  |  |  |  |  |
|  | EXP.3 | 20 ℃/Control P0 | 107.36±24.27 |  | 30 |
|  | EXP.3 | 20 ℃/HFD P0 | 350.64±48.10 | <0.0001 | 31 |
|  | EXP.3 | 20 ℃/Control F1 | 109.65±33.45 |  | 30 |
|  | EXP.3 | 20 ℃/HFD F1 | 219.67±21.40 | <0.0001 | 31 |
|  | EXP.3 | 20 ℃/Control F2 | 122.37±31.26 |  | 30 |
|  | EXP.3 | 20 ℃/HFD F2 | 158.39±21.92 | 0.0002 | 30 |
|  |  |  |  |  |  |
|  | **N2(OA)** |  |  |  |  |
| Supplementary Fig. 1b | EXP.1 | 20 ℃/Control P0 | 101.40±18.57 |  | 29 |
|  | EXP.1 | 20 ℃/OA P0 | 226.17±64.84 | <0.0001 | 29 |
|  | EXP.1 | 20 ℃/Control F1 | 101.26±25.94 |  | 30 |
|  | EXP.1 | 20 ℃/OA F1 | 142.69±26.91 | <0.0001 | 29 |
|  | EXP.1 | 20 ℃/Control F2 | 105.33±28.33 |  | 29 |
|  | EXP.1 | 20 ℃/OA F2 | 133.08±30.76 | 0.044 | 29 |
|  |  |  |  |  |  |
|  | EXP.2 | 20 ℃/Control P0 | 96.28±14.70 |  | 31 |
|  | EXP.2 | 20 ℃/OA P0 | 271.31±105.23 | <0.0001 | 31 |
|  | EXP.2 | 20 ℃/Control F1 | 111.25±25.91 |  | 31 |
|  | EXP.2 | 20 ℃/OA F1 | 154.60±30.08 | <0.0001 | 31 |
|  | EXP.2 | 20 ℃/Control F2 | 108.77±23.60 |  | 31 |
|  | EXP.2 | 20 ℃/OA F2 | 133.14±24.85 | 0.014 | 31 |
|  |  |  |  |  |  |
|  | EXP.3 | 20 ℃/Control P0 | 102.61±27.29 |  | 29 |
|  | EXP.3 | 20 ℃/OA P0 | 243.99±79.17 | <0.0001 | 30 |
|  | EXP.3 | 20 ℃/Control F1 | 111.88±24.69 |  | 30 |
|  | EXP.3 | 20 ℃/OA F1 | 154.55±29.62 | <0.0001 | 31 |
|  | EXP.3 | 20 ℃/Control F2 | 105.94±25.44 |  | 32 |
|  | EXP.3 | 20 ℃/OA F2 | 139.31±25.09 | 0.0008 | 32 |
|  |  |  |  |  |  |
| Supplementary  Fig. 1c | **N2(PA)** |  |  |  |  |
|  | EXP.1 | 20 ℃/Control P0 | 96.28±14.70 |  | 31 |
|  | EXP.1 | 20 ℃/PA P0 | 227.93±65.05 | <0.0001 | 29 |
|  | EXP.1 | 20 ℃/Control F1 | 104.04±28.63 |  | 36 |
|  | EXP.1 | 20 ℃/PA F1 | 136.51±25.16 | <0.0001 | 35 |
|  | EXP.1 | 20 ℃/Control F2 | 106.09±26.99 |  | 37 |
|  | EXP.1 | 20 ℃/PA F2 | 126.23±23.52 | 0.009 | 35 |
|  |  |  |  |  |  |
|  | EXP.2 | 20 ℃/Control P0 | 101.09±19.73 |  | 33 |
|  | EXP.2 | 20 ℃/PA P0 | 243.20±94.65 | <0.0001 | 32 |
|  | EXP.2 | 20 ℃/Control F1 | 104.97±25.84 |  | 34 |
|  | EXP.2 | 20 ℃/PA F1 | 157.08±25.33 | <0.0001 | 29 |
|  | EXP.2 | 20 ℃/Control F2 | 102.78±24.04 |  | 30 |
|  | EXP.2 | 20 ℃/PA F2 | 132.86±25.31 | 0.005 | 29 |
|  |  |  |  |  |  |
|  | EXP.3 | 20 ℃/Control P0 | 101.69±25.25 |  | 31 |
|  | EXP.3 | 20 ℃/PA P0 | 233.01±66.46 | <0.0001 | 29 |
|  | EXP.3 | 20 ℃/Control F1 | 108.51±36.50 |  | 35 |
|  | EXP.3 | 20 ℃/PA F1 | 134.46±23.04 | <0.0001 | 35 |
|  | EXP.3 | 20 ℃/Control F2 | 103.21±26.58 |  | 36 |
|  | EXP.3 | 20 ℃/PA F2 | 134.90±17.13 | 0.004 | 34 |
|  |  |  |  |  |  |
|  | **CE541 *sbp-1(ep79)*** | |  |  |  |
| Fig. 2a | **N2** | **20 ℃/Control P0** | **111.20±29.46** |  | **31** |
|  | **N2** | **20 ℃/HFD P0** | **260.99±102.05** | **<0.0001** | **29** |
|  |  |  |  |  |  |
|  | EXP.1 | 20 ℃/Control P0 | 13.22±6.90 |  | 30 |
|  | EXP.1 | 20 ℃/HFD P0 | 132.03±34.99 | <0.0001 | 30 |
|  | EXP.1 | 20 ℃/Control F1 | 10.58±6.38 |  | 28 |
|  | EXP.1 | 20 ℃/HFD F1 | 14.17±8.74 | 0.1005 | 28 |
|  | EXP.1 | 20 ℃/Control F2 | 11.85±7.73 |  | 29 |
|  | EXP.1 | 20 ℃/HFD F2 | 15.11±9.22 | 0.4012 | 29 |
|  |  |  |  |  |  |
|  | EXP.2 | 20 ℃/Control P0 | 10.43±5.51 |  | 32 |
|  | EXP.2 | 20 ℃/HFD P0 | 81.32±18.97 | <0.0001 | 32 |
|  | EXP.2 | 20 ℃/Control F1 | 11.42±5.25 |  | 33 |
|  | EXP.2 | 20 ℃/HFD F1 | 12.71±5.70 | 0.6465 | 33 |
|  | EXP.2 | 20 ℃/Control F2 | 12.05±5.68 |  | 33 |
|  | EXP.2 | 20 ℃/HFD F2 | 11.76±5.30 | 0.6920 | 33 |
|  |  |  |  |  |  |
|  | EXP.3 | 20 ℃/Control P0 | 12.52±5.91 |  | 36 |
|  | EXP.3 | 20 ℃/HFD P0 | 106.01±41.03 | <0.0001 | 36 |
|  | EXP.3 | 20 ℃/Control F1 | 11.35±5.38 |  | 36 |
|  | EXP.3 | 20 ℃/HFD F1 | 14.55±7.47 | 0.383 | 39 |
|  | EXP.3 | 20 ℃/Control F2 | 13.97±7.17 |  | 39 |
|  | EXP.3 | 20 ℃/HFD F2 | 14.66±7.96 | 0.961 | 39 |
|  |  |  |  |  |  |
|  | **STE70 *nhr-80(tm1011)*** | |  |  |  |
| Fig. 2b | **N2** | **20 ℃/Control P0** | **111.20±29.46** |  | **31** |
|  | **N2** | **20 ℃/HFD P0** | **260.99±102.05** | **<0.0001** | **29** |
|  |  |  |  |  |  |
|  | EXP.1 | 20 ℃/Control P0 | 139.02±38.52 |  | 31 |
|  | EXP.1 | 20 ℃/HFD P0 | 363.12±63.81 | 0.0072 | 31 |
|  | EXP.1 | 20 ℃/Control F1 | 151.19±55.49 |  | 33 |
|  | EXP.1 | 20 ℃/HFD F1 | 165.01±57.37 | 0.8518 | 33 |
|  | EXP.1 | 20 ℃/Control F2 | 133.46±44.06 |  | 29 |
|  | EXP.1 | 20 ℃/HFD F2 | 154.24±55.23 | 0.2274 | 29 |
|  |  |  |  |  |  |
|  | EXP.2 | 20 ℃/Control P0 | 155.93±31.73 |  | 31 |
|  | EXP.2 | 20 ℃/HFD P0 | 377.97±62.49 | 0.0004 | 31 |
|  | EXP.2 | 20 ℃/Control F1 | 154.25±41.71 |  | 30 |
|  | EXP.2 | 20 ℃/HFD F1 | 149.31±34.13 | 0.2863 | 30 |
|  | EXP.2 | 20 ℃/Control F2 | 139.02±38.52 |  | 31 |
|  | EXP.2 | 20 ℃/HFD F2 | 158.40±49.61 | 0.089 | 32 |
|  |  |  |  |  |  |
|  | EXP.3 | 20 ℃/Control P0 | 141.53±39.33 |  | 36 |
|  | EXP.3 | 20 ℃/HFD P0 | 372.88±67.01 | <0.0001 | 36 |
|  | EXP.3 | 20 ℃/Control F1 | 154.98±42.34 |  | 35 |
|  | EXP.3 | 20 ℃/HFD F1 | 150.55±35.77 | 0.635 | 36 |
|  | EXP.3 | 20 ℃/Control F2 | 141.09±41.71 |  | 35 |
|  | EXP.3 | 20 ℃/HFD F2 | 159.00±50.02 | 0.468 | 34 |
|  |  |  |  |  |  |
|  | **VC870 *nhr-49*(*gk405*)** | |  |  |  |
| Fig. 2c | **N2** | **20 ℃/Control P0** | **111.20±29.46** |  | **31** |
|  | **N2** | **20 ℃/HFD P0** | **260.99±102.05** | **<0.0001** | **29** |
|  |  |  |  |  |  |
|  | EXP.1 | 20 ℃/Control P0 | 167.76±40.30 |  | 31 |
|  | EXP.1 | 20 ℃/HFD P0 | 401.72±87.78 | <0.0001 | 31 |
|  | EXP.1 | 20 ℃/Control F1 | 186.12±56.07 |  | 32 |
|  | EXP.1 | 20 ℃/HFD F1 | 215.37±38.91 | 0.1434 | 33 |
|  | EXP.1 | 20 ℃/Control F2 | 167.31±29.09 |  | 32 |
|  | EXP.1 | 20 ℃/HFD F2 | 178.37±51.11 | 0.1638 | 32 |
|  |  |  |  |  |  |
|  | EXP.2 | 20 ℃/Control P0 | 180.25±58.29 |  | 31 |
|  | EXP.2 | 20 ℃/HFD P0 | 401.72±87.78 | <0.0001 | 31 |
|  | EXP.2 | 20 ℃/Control F1 | 183.35±36.59 |  | 33 |
|  | EXP.2 | 20 ℃/HFD F1 | 204.76±42.74 | 0.3838 | 33 |
|  | EXP.2 | 20 ℃/Control F2 | 169.57±28.03 |  | 31 |
|  | EXP.2 | 20 ℃/HFD F2 | 179.22±22.82 | 0.2260 | 31 |
|  |  |  |  |  |  |
|  | EXP.3 | 20 ℃/Control P0 | 190.03±56.79 |  | 35 |
|  | EXP.3 | 20 ℃/HFD P0 | 425.14±149.31 | <0.0001 | 34 |
|  | EXP.3 | 20 ℃/Control F1 | 178.57±43.76 |  | 37 |
|  | EXP.3 | 20 ℃/HFD F1 | 194.98±41.63 | 0.093 | 37 |
|  | EXP.3 | 20 ℃/Control F2 | 170.00±30.47 |  | 35 |
|  | EXP.3 | 20 ℃/HFD F2 | 265.93±36.87 | 0.925 | 37 |
|  |  |  |  |  |  |
|  | **CF1038 *daf-16(mu86)*** | |  |  |  |
| Fig. 2d | **N2** | **20 ℃/Control P0** | **111.20±29.46** |  | **31** |
|  | **N2** | **20 ℃/HFD P0** | **260.99±102.05** | **<0.0001** | **29** |
|  |  |  |  |  |  |
|  | EXP.1 | 20 ℃/Control P0 | 107.85±32.23 |  | 27 |
|  | EXP.1 | 20 ℃/HFD P0 | 119.30±36.45 | 0.5350 | 27 |
|  | EXP.1 | 20 ℃/Control F1 | 105.93±35.71 |  | 29 |
|  | EXP.1 | 20 ℃/HFD F1 | 117.55±39.12 | 0.6328 | 29 |
|  | EXP.1 | 20 ℃/Control F2 | 115.22±32.95 |  | 35 |
|  | EXP.1 | 20 ℃/HFD F2 | 121.50±25.83 | 0.3646 | 33 |
|  |  |  |  |  |  |
|  | EXP.2 | 20 ℃/Control P0 | 144.45±16.04 |  | 31 |
|  | EXP.2 | 20 ℃/HFD P0 | 147.84±44.76 | 0.8732 | 33 |
|  | EXP.2 | 20 ℃/Control F1 | 117.83±39.61 |  | 31 |
|  | EXP.2 | 20 ℃/HFD F1 | 113.92±34.90 | 0.4929 | 31 |
|  | EXP.2 | 20 ℃/Control F2 | 117.50±33.10 |  | 30 |
|  | EXP.2 | 20 ℃/HFD F2 | 126.16±37.77 | 0.1665 | 30 |
|  |  |  |  |  |  |
|  | EXP.3 | 20 ℃/Control P0 | 129.50±39.29 |  | 36 |
|  | EXP.3 | 20 ℃/HFD P0 | 135.96±47.14 | 0.404 | 36 |
|  | EXP.3 | 20 ℃/Control F1 | 108.32±28.36 |  | 30 |
|  | EXP.3 | 20 ℃/HFD F1 | 112.66±35.67 | 0.603 | 30 |
|  | EXP.3 | 20 ℃/Control F2 | 136.64±36.54 |  | 35 |
|  | EXP.3 | 20 ℃/HFD F2 | 142.80±30.34 | 0.537 | 31 |
|  |  | |  |  |  |
|  | **BX106 *fat-6(tm331)*** | |  |  |  |
| Fig. 3c | **N2** | **20 ℃/Control P0** | **136.66±37.26** |  | **31** |
|  | **N2** | **20 ℃/HFD P0** | **245.47±53.07** | **<0.0001** | **31** |
|  |  |  |  |  |  |
|  | EXP.1 | 20 ℃/Control P0 | 98.66±21.00 |  | 29 |
|  | EXP.1 | 20 ℃/HFD P0 | 230.13±53.23 | <0.0001 | 29 |
|  | EXP.1 | 20 ℃/Control F1 | 102.61±22.23 |  | 31 |
|  | EXP.1 | 20 ℃/HFD F1 | 110.91±26.94 | 0.222 | 30 |
|  | EXP.1 | 20 ℃/Control F2 | 116.88±31.43 |  | 30 |
|  | EXP.1 | 20 ℃/HFD F2 | 114.48±36.26 | 0.836 | 30 |
|  |  |  |  |  |  |
|  | EXP.2 | 20 ℃/Control P0 | 97.54±20.58 |  | 33 |
|  | EXP.2 | 20 ℃/HFD P0 | 230.31±56.15 | <0.0001 | 32 |
|  | EXP.2 | 20 ℃/Control F1 | 98.66±21.00 |  | 29 |
|  | EXP.2 | 20 ℃/HFD F1 | 114.92±23.82 | 0.069 | 29 |
|  | EXP.2 | 20 ℃/Control F2 | 105.78±31.35 |  | 36 |
|  | EXP.2 | 20 ℃/HFD F2 | 110.95±30.31 | 0.902 | 38 |
|  |  |  |  |  |  |
|  | EXP.3 | 20 ℃/Control P0 | 99.45±20.90 |  | 33 |
|  | EXP.3 | 20 ℃/HFD P0 | 230.93±52.94 | <0.0001 | 33 |
|  | EXP.3 | 20 ℃/Control F1 | 98.83±25.00 |  | 30 |
|  | EXP.3 | 20 ℃/HFD F1 | 110.68±31.29 | 0.061 | 31 |
|  | EXP.3 | 20 ℃/Control F2 | 102.87±20.78 |  | 32 |
|  | EXP.3 | 20 ℃/HFD F2 | 106.67±28.17 | 0.516 | 31 |
|  |  |  |  |  |  |
|  | **BX107 *fat-5(tm420)*** | |  |  |  |
| Fig. 3b | **N2** | **20 ℃/Control P0** | **136.66±37.26** |  | **31** |
|  | **N2** | **20 ℃/HFD P0** | **245.47±53.07** | **<0.0001** | **31** |
|  |  |  |  |  |  |
|  | EXP.1 | 20 ℃/Control P0 | 116.89±25.99 |  | 32 |
|  | EXP.1 | 20 ℃/HFD P0 | 300.71±57.70 | <0.0001 | 32 |
|  | EXP.1 | 20 ℃/Control F1 | 97.06±37.07 |  | 32 |
|  | EXP.1 | 20 ℃/HFD F1 | 104.89±26.95 | 0.882 | 32 |
|  | EXP.1 | 20 ℃/Control F2 | 105.00±32.12 |  | 32 |
|  | EXP.1 | 20 ℃/HFD F2 | 105.51±26.95 | 0.5950 | 32 |
|  |  |  |  |  |  |
|  | EXP.2 | 20 ℃/Control P0 | 109.43±24.10 |  | 29 |
|  | EXP.2 | 20 ℃/HFD P0 | 299.82±70.62 | 0.0001 | 29 |
|  | EXP.2 | 20 ℃/Control F1 | 97.06±37.07 |  | 32 |
|  | EXP.2 | 20 ℃/HFD F1 | 104.51±26.95 | 0.0808 | 32 |
|  | EXP.2 | 20 ℃/Control F2 | 104.03±26.94 |  | 37 |
|  | EXP.2 | 20 ℃/HFD F2 | 95.97±26.48 | 0.091 | 37 |
|  |  |  |  |  |  |
|  | EXP.3 | 20 ℃/Control P0 | 104.14±34.32 |  | 33 |
|  | EXP.3 | 20 ℃/HFD P0 | 311.37±77.82 | 0.0001 | 33 |
|  | EXP.3 | 20 ℃/Control F1 | 98.13±31.53 |  | 34 |
|  | EXP.3 | 20 ℃/HFD F1 | 101.85±27.16 | 0.963 | 35 |
|  | EXP.3 | 20 ℃/Control F2 | 107.28±27.44 |  | 36 |
|  | EXP.3 | 20 ℃/HFD F2 | 101.11±27.21 | 0.422 | 34 |
|  |  |  |  |  |  |
|  | **BX110 *fat-5(tm420); fat-6(tm331)*** | | |  |  |
| Fig. 3e | **N2** | **20 ℃/Control P0** | **136.66±37.26** |  | **31** |
|  | **N2** | **20 ℃/HFD P0** | **245.47±53.07** | **<0.0001** | **31** |
|  |  |  |  |  |  |
|  | EXP.1 | 20 ℃/Control P0 | 104.89±22.59 |  | 30 |
|  | EXP.1 | 20 ℃/HFD P0 | 207.44±57.90 | <0.0001 | 30 |
|  | EXP.1 | 20 ℃/Control F1 | 105.89±21.63 |  | 33 |
|  | EXP.1 | 20 ℃/HFD F1 | 104.37±23.31 | 0.636 | 33 |
|  | EXP.1 | 20 ℃/Control F2 | 106.90±20.74 |  | 30 |
|  | EXP.1 | 20 ℃/HFD F2 | 104.74±20.28 | 0.909 | 30 |
|  |  |  |  |  |  |
|  | EXP.2 | 20 ℃/Control P0 | 94.10±28.73 |  | 28 |
|  | EXP.2 | 20 ℃/HFD P0 | 231.47±58.22 | <0.0001 | 28 |
|  | EXP.2 | 20 ℃/Control F1 | 105.89±21.63 |  | 33 |
|  | EXP.2 | 20 ℃/HFD F1 | 104.37±23.31 | 0.912 | 33 |
|  | EXP.2 | 20 ℃/Control F2 | 96.61±18.91 |  | 31 |
|  | EXP.2 | 20 ℃/HFD F2 | 93.23±21.46 | 0.325 | 31 |
|  |  |  |  |  |  |
|  | EXP.3 | 20 ℃/Control P0 | 96.61±26.79 |  | 31 |
|  | EXP.3 | 20 ℃/HFD P0 | 217.83±63.51 | <0.0001 | 31 |
|  | EXP.3 | 20 ℃/Control F1 | 104.00±20.20 |  | 31 |
|  | EXP.3 | 20 ℃/HFD F1 | 107.05±24.35 | 0.677 | 30 |
|  | EXP.3 | 20 ℃/Control F2 | 103.20±19.52 |  | 32 |
|  | EXP.3 | 20 ℃/HFD F2 | 100.14±19.56 | 0.516 | 34 |
|  |  |  |  |  |  |
|  | **BX153 *fat-7(wa36)*** | |  |  |  |
| Fig. 3d | **N2** | **20 ℃/Control P0** | **136.66±37.26** |  | **31** |
|  | **N2** | **20 ℃/HFD P0** | **245.47±53.07** | **<0.0001** | **31** |
|  |  |  |  |  |  |
|  | EXP.1 | 20 ℃/Control P0 | 100.23±22.35 |  | 30 |
|  | EXP.1 | 20 ℃/HFD P0 | 218.37±58.93 | <0.0001 | 35 |
|  | EXP.1 | 20 ℃/Control F1 | 102.55±25.51 |  | 34 |
|  | EXP.1 | 20 ℃/HFD F1 | 115.86±29.81 | 0.111 | 31 |
|  | EXP.1 | 20 ℃/Control F2 | 116.23±31.78 |  | 29 |
|  | EXP.1 | 20 ℃/HFD F2 | 114.48±36.26 | 0.394 | 30 |
|  |  |  |  |  |  |
|  | EXP.2 | 20 ℃/Control P0 | 118.79±34.80 |  | 34 |
|  | EXP.2 | 20 ℃/HFD P0 | 278.78±57.72 | <0.0001 | 35 |
|  | EXP.2 | 20 ℃/Control F1 | 102.31±29.67 |  | 30 |
|  | EXP.2 | 20 ℃/HFD F1 | 109.40±28.27 | 0.269 | 30 |
|  | EXP.2 | 20 ℃/Control F2 | 109.01±32.33 |  | 34 |
|  | EXP.2 | 20 ℃/HFD F2 | 116.28±26.08 | 0.413 | 34 |
|  |  |  |  |  |  |
|  | EXP.3 | 20 ℃/Control P0 | 119.43±37.99 |  | 36 |
|  | EXP.3 | 20 ℃/HFD P0 | 281.69±61.79 | <0.0001 | 36 |
|  | EXP.3 | 20 ℃/Control F1 | 106.50±28.82 |  | 37 |
|  | EXP.3 | 20 ℃/HFD F1 | 107.67±31.71 | 0.984 | 35 |
|  | EXP.3 | 20 ℃/Control F2 | 114.59±36.68 |  | 38 |
|  | EXP.3 | 20 ℃/HFD F2 | 116.35±22.75 | 0.874 | 39 |
|  |  |  |  |  |  |
|  | **BX160 *fat-5(tm420); fat-7(wa36)*** | |  |  |  |
| Fig. 3f | **N2** | **20 ℃/Control P0** | **136.66±37.26** |  | **31** |
|  | **N2** | **20 ℃/HFD P0** | **245.47±53.07** | **<0.0001** | **31** |
|  |  |  |  |  |  |
|  | EXP.1 | 20 ℃/Control P0 | 131.86±24.78 |  | 30 |
|  | EXP.1 | 20 ℃/HFD P0 | 289.88±46.52 | <0.0001 | 30 |
|  | EXP.1 | 20 ℃/Control F1 | 105.09±26.83 |  | 31 |
|  | EXP.1 | 20 ℃/HFD F1 | 103.74±25.02 | 0.198 | 31 |
|  | EXP.1 | 20 ℃/Control F2 | 104.76±19.98 |  | 30 |
|  | EXP.1 | 20 ℃/HFD F2 | 113.54±27.31 | 0.083 | 30 |
|  |  |  |  |  |  |
|  | EXP.2 | 20 ℃/Control P0 | 101.81±26.31 |  | 30 |
|  | EXP.2 | 20 ℃/HFD P0 | 347.36±90.58 | <0.0001 | 31 |
|  | EXP.2 | 20 ℃/Control F1 | 105.09±26.83 |  | 31 |
|  | EXP.2 | 20 ℃/HFD F1 | 103.74±25.02 | 0.828 | 31 |
|  | EXP.2 | 20 ℃/Control F2 | 105.39±26.88 |  | 32 |
|  | EXP.2 | 20 ℃/HFD F2 | 109.19±21.28 | 0.352 | 31 |
|  |  |  |  |  |  |
|  | EXP.3 | 20 ℃/Control P0 | 113.67±29.68 |  | 33 |
|  | EXP.3 | 20 ℃/HFD P0 | 316.05±84.13 | <0.0001 | 33 |
|  | EXP.3 | 20 ℃/Control F1 | 114.18±30.56 |  | 35 |
|  | EXP.3 | 20 ℃/HFD F1 | 115.00±32.20 | 0.674 | 36 |
|  | EXP.3 | 20 ℃/Control F2 | 101.38±24.29 |  | 35 |
|  | EXP.3 | 20 ℃/HFD F2 | 115.98±27.73 | 0.121 | 36 |
|  |  |  |  |  |  |
|  | **RB1304 *wdr-5.1(ok1417)*** | |  |  |  |
| Fig. 4a | **N2** | **20 ℃/Control P0** | **111.04±27.11** |  | **31** |
|  | **N2** | **20 ℃/HFD P0** | **278.11±87.86** | **<0.0001** | **31** |
|  |  |  |  |  |  |
|  | EXP.1 | 20 ℃/Control P0 | 214.50±34.67 |  | 29 |
|  | EXP.1 | 20 ℃/HFD P0 | 408.24±75.37 | <0.0001 | 29 |
|  | EXP.1 | 20 ℃/Control F1 | 218.85±41.07 |  | 38 |
|  | EXP.1 | 20 ℃/HFD F1 | 215.49±42.38 | 0.86 | 38 |
|  | EXP.1 | 20 ℃/Control F2 | 208.19±35.99 |  | 28 |
|  | EXP.1 | 20 ℃/HFD F2 | 188.51±36.12 | 0.432 | 28 |
|  |  |  |  |  |  |
|  | EXP.2 | 20 ℃/Control P0 | 197.93±38.79 |  | 30 |
|  | EXP.2 | 20 ℃/HFD P0 | 434.01±81.94 | <0.0001 | 30 |
|  | EXP.2 | 20 ℃/Control F1 | 227.06±40.61 |  | 30 |
|  | EXP.2 | 20 ℃/HFD F1 | 221.04±48.98 | 0.672 | 33 |
|  | EXP.2 | 20 ℃/Control F2 | 227.63±44.70 |  | 34 |
|  | EXP.2 | 20 ℃/HFD F2 | 221.38±42.44 | 0.284 | 33 |
|  |  |  |  |  |  |
|  | EXP.3 | 20 ℃/Control P0 | 207.93±52.43 |  | 30 |
|  | EXP.3 | 20 ℃/HFD P0 | 434.01±81.94 | <0.0001 | 30 |
|  | EXP.3 | 20 ℃/Control F1 | 222.36±56.21 |  | 30 |
|  | EXP.3 | 20 ℃/HFD F1 | 221.04±48.98 | 0.726 | 33 |
|  | EXP.3 | 20 ℃/Control F2 | 224.44±44.34 |  | 31 |
|  | EXP.3 | 20 ℃/HFD F2 | 227.21±35.71 | 0.535 | 30 |
|  |  |  |  |  |  |
|  | **ZR2 *jmjd-3.1(gk384)*** | |  |  |  |
| Supplementary Fig. 6c | **N2** | **20 ℃/Control P0** | **111.04±27.11** |  | **31** |
|  | **N2** | **20 ℃/HFD P0** | **278.11±87.86** | **<0.0001** | **31** |
|  |  |  |  |  |  |
|  | EXP.1 | 20 ℃/Control P0 | 113.13±25.05 |  | 30 |
|  | EXP.1 | 20 ℃/HFD P0 | 227.34±59.56 | <0.0001 | 30 |
|  | EXP.1 | 20 ℃/Control F1 | 120.50±36.89 |  | 30 |
|  | EXP.1 | 20 ℃/HFD F1 | 199.17±51.52 | <0.0001 | 30 |
|  | EXP.1 | 20 ℃/Control F2 | 125.22±26.89 |  | 30 |
|  | EXP.1 | 20 ℃/HFD F2 | 182.51±46.66 | 0.019 | 30 |
|  |  |  |  |  |  |
|  | EXP.2 | 20 ℃/Control P0 | 108.47±26.67 |  | 31 |
|  | EXP.2 | 20 ℃/HFD P0 | 218.60±45.82 | <0.0001 | 31 |
|  | EXP.2 | 20 ℃/Control F1 | 113.16±26.69 |  | 30 |
|  | EXP.2 | 20 ℃/HFD F1 | 205.19±42.45 | <0.0001 | 30 |
|  | EXP.2 | 20 ℃/Control F2 | 108.60±27.66 |  | 32 |
|  | EXP.2 | 20 ℃/HFD F2 | 206.64±40.48 | <0.0001 | 32 |
|  |  |  |  |  |  |
|  | EXP.3 | 20 ℃/Control P0 | 120.33±21.94 |  | 29 |
|  | EXP.3 | 20 ℃/HFD P0 | 226.67±51.07 | <0.0001 | 29 |
|  | EXP.3 | 20 ℃/Control F1 | 110.60±32.38 |  | 29 |
|  | EXP.3 | 20 ℃/HFD F1 | 191.58±37.30 | <0.0001 | 29 |
|  | EXP.3 | 20 ℃/Control F2 | 115.25±32.56 |  | 29 |
|  | EXP.3 | 20 ℃/HFD F2 | 162.48±61.84 | 0.036 | 29 |
|  |  |  |  |  |  |
| Supplementary  Fig. 6d | **VC1666 *met-1(ok2172)*** | |  |  |  |
|  | **N2** | **20 ℃/Control P0** | **111.04±27.11** |  | **31** |
|  | **N2** | **20 ℃/HFD P0** | **278.11±87.86** | **<0.0001** | **31** |
|  |  |  |  |  |  |
|  | EXP.1 | 20 ℃/Control P0 | 128.44±56.66 |  | 30 |
|  | EXP.1 | 20 ℃/HFD P0 | 431.57±83.85 | <0.0001 | 30 |
|  | EXP.1 | 20 ℃/Control F1 | 99.57±28.38 |  | 30 |
|  | EXP.1 | 20 ℃/HFD F1 | 173.26±34.49 | <0.0001 | 30 |
|  | EXP.1 | 20 ℃/Control F2 | 91.59±23.43 |  | 30 |
|  | EXP.1 | 20 ℃/HFD F2 | 146.64±17.84 | <0.0001 | 30 |
|  |  |  |  |  |  |
|  | EXP.2 | 20 ℃/Control P0 | 116.62±47.66 |  | 35 |
|  | EXP.2 | 20 ℃/HFD P0 | 407.25±84.37 | <0.0001 | 35 |
|  | EXP.2 | 20 ℃/Control F1 | 101.81±31.30 |  | 34 |
|  | EXP.2 | 20 ℃/HFD F1 | 153.62±50.51 | 0.0008 | 34 |
|  | EXP.2 | 20 ℃/Control F2 | 97.14±25.98 |  | 33 |
|  | EXP.2 | 20 ℃/HFD F2 | 161.79±41.34 | <0.0001 | 35 |
|  |  |  |  |  |  |
|  | EXP.3 | 20 ℃/Control P0 | 139.53±80.87 |  | 31 |
|  | EXP.3 | 20 ℃/HFD P0 | 407.22±88.61 | <0.0001 | 34 |
|  | EXP.3 | 20 ℃/Control F1 | 133.66±58.99 |  | 34 |
|  | EXP.3 | 20 ℃/HFD F1 | 203.99±63.99 | 0.0002 | 33 |
|  | EXP.3 | 20 ℃/Control F2 | 124.79±47.64 |  | 34 |
|  | EXP.3 | 20 ℃/HFD F2 | 176.06±62.13 | 0.042 | 33 |
|  |  |  |  |  |  |
| Supplementary  Fig. 6a | **tm1726 *ash-2(tm1726)*** | |  |  |  |
|  | **N2** | **20 ℃/Control P0** | **128.09±38.87** |  | **32** |
|  | **N2** | **20 ℃/HFD P0** | **314.48±97.07** | **<0.0001** | **33** |
|  |  |  |  |  |  |
|  | EXP.1 | 20 ℃/Control P0 | 150.97±48.79 |  | 30 |
|  | EXP.1 | 20 ℃/HFD P0 | 484.82±85.15 | <0.0001 | 30 |
|  | EXP.1 | 20 ℃/Control F1 | 164.80±40.15 |  | 31 |
|  | EXP.1 | 20 ℃/HFD F1 | 293.81±41.07 | <0.0001 | 31 |
|  | EXP.1 | 20 ℃/Control F2 | 160.57±37.15 |  | 31 |
|  | EXP.1 | 20 ℃/HFD F2 | 226.59±28.36 | <0.0001 | 30 |
|  |  |  |  |  |  |
|  | EXP.2 | 20 ℃/Control P0 | 157.72±57.03 |  | 30 |
|  | EXP.2 | 20 ℃/HFD P0 | 488.54±95.83 | <0.0001 | 30 |
|  | EXP.2 | 20 ℃/Control F1 | 153.39±32.72 |  | 30 |
|  | EXP.2 | 20 ℃/HFD F1 | 277.93±38.67 | <0.0001 | 31 |
|  | EXP.2 | 20 ℃/Control F2 | 153.29±33.95 |  | 31 |
|  | EXP.2 | 20 ℃/HFD F2 | 221.26±29.16 | <0.0001 | 31 |
|  |  |  |  |  |  |
|  | EXP.3 | 20 ℃/Control P0 | 147.45±38.70 |  | 30 |
|  | EXP.3 | 20 ℃/HFD P0 | 481.21±100.39 | <0.0001 | 30 |
|  | EXP.3 | 20 ℃/Control F1 | 161.19±35.45 |  | 30 |
|  | EXP.3 | 20 ℃/HFD F1 | 283.84±33.95 | <0.0001 | 30 |
|  | EXP.3 | 20 ℃/Control F2 | 159.80±40.88 |  | 30 |
|  | EXP.3 | 20 ℃/HFD F2 | 213.29±29.80 | <0.0001 | 31 |
|  |  |  |  |  |  |
| Supplementary  Fig. 6b | **VC974 *set-2(ok1484)*** | |  |  |  |
|  | **N2** | **20 ℃/Control P0** | **128.09±38.87** |  | **32** |
|  | **N2** | **20 ℃/HFD P0** | **314.48±97.07** | **<0.0001** | **33** |
|  |  |  |  |  |  |
|  | EXP.1 | 20 ℃/Control P0 | 155.68±45.37 |  | 30 |
|  | EXP.1 | 20 ℃/HFD P0 | 470.04±108.42 | <0.0001 | 30 |
|  | EXP.1 | 20 ℃/Control F1 | 152.70±44.97 |  | 30 |
|  | EXP.1 | 20 ℃/HFD F1 | 285.43±38.44 | <0.0001 | 30 |
|  | EXP.1 | 20 ℃/Control F2 | 141.38±41.46 |  | 30 |
|  | EXP.1 | 20 ℃/HFD F2 | 234.95±38.77 | <0.0001 | 30 |
|  |  |  |  |  |  |
|  | EXP.2 | 20 ℃/Control P0 | 150.16±40.79 |  | 30 |
|  | EXP.2 | 20 ℃/HFD P0 | 439.71±96.93 | <0.0001 | 30 |
|  | EXP.2 | 20 ℃/Control F1 | 142.77±46.99 |  | 30 |
|  | EXP.2 | 20 ℃/HFD F1 | 279.40±44.29 | <0.0001 | 30 |
|  | EXP.2 | 20 ℃/Control F2 | 151.50±48.01 |  | 30 |
|  | EXP.2 | 20 ℃/HFD F2 | 219.30±11.26 | <0.0001 | 30 |
|  |  |  |  |  |  |
|  | EXP.3 | 20 ℃/Control P0 | 149.54±43.82 |  | 31 |
|  | EXP.3 | 20 ℃/HFD P0 | 447.22±115.98 | <0.0001 | 30 |
|  | EXP.3 | 20 ℃/Control F1 | 146.06±39.26 |  | 30 |
|  | EXP.3 | 20 ℃/HFD F1 | 269.55±39.92 | <0.0001 | 30 |
|  | EXP.3 | 20 ℃/Control F2 | 161.71±34.37 |  | 30 |
|  | EXP.3 | 20 ℃/HFD F2 | 216.24±11.37 | <0.0001 | 30 |
|  |  |  |  |  |  |
|  | **TU3401 Neuron specific *daf-16* RNAi** | |  |  |  |
| Fig. 5h | EXP.1 | 20 ℃/Control P0 | 23.62±9.96 |  | 30 |
|  | EXP.1 | 20 ℃/HFD P0 | 92.17±25.91 | <0.0001 | 30 |
|  | EXP.2 | 20 ℃/Control P0 | 23.83±9.45 |  | 31 |
|  | EXP.2 | 20 ℃/HFD P0 | 93.68±26.39 | <0.0001 | 31 |
|  | EXP.3 | 20 ℃/Control P0 | 22.66±11.42 |  | 32 |
|  | EXP.3 | 20 ℃/HFD P0 | 96.73±28.22 | <0.0001 | 32 |
|  |  |  |  |  |  |
|  | **VP303 Intestine specific *daf-16* RNAi** | |  |  |  |
| Fig. 5i | EXP.1 | 20 ℃/Control P0 | 52.03±16.02 |  | 30 |
|  | EXP.1 | 20 ℃/HFD P0 | 126.47±25.22 | <0.0001 | 30 |
|  | EXP.2 | 20 ℃/Control P0 | 45.10±11.76 |  | 31 |
|  | EXP.2 | 20 ℃/HFD P0 | 145.27±36.90 | <0.0001 | 31 |
|  | EXP.3 | 20 ℃/Control P0 | 47.07±13.45 |  | 34 |
|  | EXP.3 | 20 ℃/HFD P0 | 134.04±31.43 | <0.0001 | 35 |
|  |  | | |  |  |
|  | **AMJ345 Germline and intestine specific *daf-16* RNAi** | | |  |  |
| Fig. 5j | EXP.1 | 20 ℃/Control P0 | 50.93±15.36 |  | 30 |
|  | EXP.1 | 20 ℃/HFD P0 | 177.96±48.32 | <0.0001 | 30 |
|  | EXP.2 | 20 ℃/Control P0 | 46.87±11.87 |  | 31 |
|  | EXP.2 | 20 ℃/HFD P0 | 152.72±40.69 | <0.0001 | 31 |
|  | EXP.3 | 20 ℃/Control P0 | 52.13±15.54 |  | 34 |
|  | EXP.3 | 20 ℃/HFD P0 | 168.12±37.03 | <0.0001 | 34 |
|  |  |  |  |  |  |
|  | **MAH23 Germline specific *daf-16* RNAi** | | |  |  |
| Fig. 5k | EXP.1 | 20 ℃/Control P0 | 93.11±18.11 |  | 32 |
|  | EXP.1 | 20 ℃/HFD P0 | 180.37±41.75 | <0.0001 | 32 |
|  | EXP.2 | 20 ℃/Control P0 | 78.26±20.67 |  | 31 |
|  | EXP.2 | 20 ℃/HFD P0 | 186.18±36.41 | <0.0001 | 31 |
|  | EXP.3 | 20 ℃/Control P0 | 82.64±23.21 |  | 35 |
|  | EXP.3 | 20 ℃/HFD P0 | 178.92±37.14 | <0.0001 | 36 |
|  |  |  |  |  |  |
|  | **NR350 Muscle specific *daf-16* RNAi** | |  |  |  |
| Fig. 5g | EXP.1 | 20 ℃/Control P0 | 81.53±22.54 |  | 33 |
|  | EXP.1 | 20 ℃/HFD P0 | 95.80±26.35 | 0.139 | 33 |
|  | EXP.2 | 20 ℃/Control P0 | 82.85±31.43 |  | 31 |
|  | EXP.2 | 20 ℃/HFD P0 | 89.80±37.14 | 0.430 | 31 |
|  | EXP.3 | 20 ℃/Control P0 | 83.01±27.43 |  | 32 |
|  | EXP.3 | 20 ℃/HFD P0 | 88.34±29.39 | 0.456 | 33 |
|  | **N2 (HT115)** |  |  |  |  |
| Fig. 5l | EXP.1 | 20 ℃/Control P0 | 63.08±18.69 |  | 30 |
|  | EXP.1 | 20 ℃/HFD P0 | 253.80±83.18 | <0.0001 | 30 |
|  | EXP.2 | 20 ℃/Control P0 | 61.46±16.28 |  | 31 |
|  | EXP.2 | 20 ℃/HFD P0 | 249.49±88.22 | <0.0001 | 32 |
|  | EXP.3 | 20 ℃/Control P0 | 63.51±21.43 |  | 32 |
|  | EXP.3 | 20 ℃/HFD P0 | 254.35±91.57 | <0.0001 | 33 |
|  |  |  |  |  |  |
| Supplementary  Fig. 10b | **TU3401 Neuron specific *sbp-1* RNAi** | | |  |  |
|  | EXP.1 | 20 ℃/Control P0 | 12.85±2.05 |  | 30 |
|  | EXP.1 | 20 ℃/HFD P0 | 38.89±2.01 | <0.0001 | 30 |
|  | EXP.2 | 20 ℃/Control P0 | 11.74±1.47 |  | 30 |
|  | EXP.2 | 20 ℃/HFD P0 | 38.56±1.91 | <0.0001 | 30 |
|  | EXP.3 | 20 ℃/Control P0 | 12.70±1.36 |  | 30 |
|  | EXP.3 | 20 ℃/HFD P0 | 39.02±1.98 | <0.0001 | 30 |
|  |  |  |  |  |  |
| Supplementary  Fig. 10c | **VP303 Intestine specific *sbp-1* RNAi** | | |  |  |
|  | EXP.1 | 20 ℃/Control P0 | 25.26±2.29 |  | 30 |
|  | EXP.1 | 20 ℃/HFD P0 | 113.48±25.53 | <0.0001 | 30 |
|  | EXP.2 | 20 ℃/Control P0 | 25.80±1.28 |  | 30 |
|  | EXP.2 | 20 ℃/HFD P0 | 116.54±29.02 | <0.0001 | 30 |
|  | EXP.3 | 20 ℃/Control P0 | 26.00±2.55 |  | 30 |
|  | EXP.3 | 20 ℃/HFD P0 | 118.96±27.64 | <0.0001 | 30 |
|  |  |  |  |  |  |
| Supplementary  Fig, 10d | **AMJ345 Germline and intestine specific *sbp-1* RNAi** | | |  |  |
|  | EXP.1 | 20 ℃/Control P0 | 31.80±9.61 |  | 30 |
|  | EXP.1 | 20 ℃/HFD P0 | 123.65±15.76 | <0.0001 | 30 |
|  | EXP.2 | 20 ℃/Control P0 | 29.91±6.55 |  | 31 |
|  | EXP.2 | 20 ℃/HFD P0 | 116.09±14.87 | <0.0001 | 30 |
|  | EXP.3 | 20 ℃/Control P0 | 32.96±4.02 |  | 30 |
|  | EXP.3 | 20 ℃/HFD P0 | 118.70±15.11 | <0.0001 | 30 |
|  |  |  |  |  |  |
| Supplementary  Fig. 10e | **MAH23 Germline specific *sbp-1*RNAi** | | |  |  |
|  | EXP.1 | 20 ℃/Control P0 | 58.07±12.42 |  | 30 |
|  | EXP.1 | 20 ℃/HFD P0 | 160.28±41.44 | <0.0001 | 31 |
|  | EXP.2 | 20 ℃/Control P0 | 56.64±13.02 |  | 30 |
|  | EXP.2 | 20 ℃/HFD P0 | 157.78±25.65 | <0.0001 | 30 |
|  | EXP.3 | 20 ℃/Control P0 | 56.74±8.30 |  | 30 |
|  | EXP.3 | 20 ℃/HFD P0 | 168.76±37.64 | <0.0001 | 30 |
|  |  |  |  |  |  |
| Supplementary  Fig. 10a | **NR350 Muscle specific *sbp-1* RNAi** | | |  |  |
|  | EXP.1 | 20 ℃/Control P0 | 50.25±12.24 |  | 30 |
|  | EXP.1 | 20 ℃/HFD P0 | 101.18±11.88 | <0.0001 | 30 |
|  | EXP.2 | 20 ℃/Control P0 | 48.55±8.90 |  | 31 |
|  | EXP.2 | 20 ℃/HFD P0 | 104.81±17.24 | <0.0001 | 30 |
|  | EXP.3 | 20 ℃/Control P0 | 51.05±7.77 |  | 32 |
|  | EXP.3 | 20 ℃/HFD P0 | 106.90±14.81 | <0.0001 | 30 |
|  |  |  |  |  |  |
| Supplementary  Fig. 9d | **VP303 Intestine specific *daf-2* RNAi** | | |  |  |
|  | EXP.1 | 20 ℃/Control P0 | 75.31±19.61 |  | 30 |
|  | EXP.1 | 20 ℃/HFD P0 | 174.65±30.74 | <0.0001 | 30 |
|  | EXP.2 | 20 ℃/Control P0 | 78.41±16.23 |  | 30 |
|  | EXP.2 | 20 ℃/HFD P0 | 181.93±32.02 | <0.0001 | 30 |
|  | EXP.3 | 20 ℃/Control P0 | 79.32±18.51 |  | 30 |
|  | EXP.3 | 20 ℃/HFD P0 | 180.71±22.55 | <0.0001 | 30 |
|  |  |  |  |  |  |
| Supplementary  Fig. 9e | **AMJ345 Germline and intestine specific *daf-2* RNAi** | | |  |  |
|  | EXP.1 | 20 ℃/Control P0 | 97.49±9.08 |  | 30 |
|  | EXP.1 | 20 ℃/HFD P0 | 208.82±27.71 | <0.0001 | 30 |
|  | EXP.2 | 20 ℃/Control P0 | 98.42±9.31 |  | 30 |
|  | EXP.2 | 20 ℃/HFD P0 | 209.73±22.48 | <0.0001 | 30 |
|  | EXP.3 | 20 ℃/Control P0 | 97.69±10.08 |  | 30 |
|  | EXP.3 | 20 ℃/HFD P0 | 221.67±24.68 | <0.0001 | 30 |
|  |  |  |  |  |  |
| Supplementary  Fig. 9f | **MAH23 Germline specific *daf-2* RNAi** | | |  |  |
|  | EXP.1 | 20 ℃/Control P0 | 135.93±36.79 |  | 30 |
|  | EXP.1 | 20 ℃/HFD P0 | 247.92±24.44 | <0.0001 | 30 |
|  | EXP.2 | 20 ℃/Control P0 | 133.91±23.92 |  | 31 |
|  | EXP.2 | 20 ℃/HFD P0 | 262.42±21.03 | <0.0001 | 30 |
|  | EXP.3 | 20 ℃/Control P0 | 135.39±27.33 |  | 30 |
|  | EXP.3 | 20 ℃/HFD P0 | 255.17±20.21 | <0.0001 | 30 |
|  |  |  |  |  |  |
|  | ***daf-16* (OE)*; nhr-49* (lof)** | |  |  |  |
| Supplementary Fig. 3a | EXP.1 | 20 ℃/Control P0 | 161.75±25.89 |  | 31 |
|  | EXP.1 | 20 ℃/HFD P0 | 365.97±29.28 | <0.0001 | 30 |
|  | EXP.1 | 20 ℃/Control F1 | 167.33±24.57 |  | 30 |
|  | EXP.1 | 20 ℃/HFD F1 | 161.85±26.33 | 0.408 | 30 |
|  |  |  |  |  |  |
|  | EXP.2 | 20 ℃/Control P0 | 161.59±22.47 |  | 30 |
|  | EXP.2 | 20 ℃/HFD P0 | 367.81±31.71 | <0.0001 | 30 |
|  | EXP.2 | 20 ℃/Control F1 | 172.57±21.99 |  | 30 |
|  | EXP.2 | 20 ℃/HFD F1 | 173.42±21.99 | 0.882 | 30 |
|  |  |  |  |  |  |
|  | EXP.3 | 20 ℃/Control P0 | 169.88±33.14 |  | 30 |
|  | EXP.3 | 20 ℃/HFD P0 | 374.98±57.68 | <0.0001 | 30 |
|  | EXP.3 | 20 ℃/Control F1 | 173.54±18.95 |  | 30 |
|  | EXP.3 | 20 ℃/HFD F1 | 168.09±22.98 | 0.321 | 30 |
|  |  |  |  |  |  |
| Supplementary  Fig. 3b | ***daf-16* (OE)*; nhr-80* (lof)** | |  |  |  |
|  | EXP.1 | 20 ℃/Control P0 | 163.61±8.19 |  | 31 |
|  | EXP.1 | 20 ℃/HFD P0 | 370.78±10.80 | <0.0001 | 30 |
|  | EXP.1 | 20 ℃/Control F1 | 161.30±7.50 |  | 30 |
|  | EXP.1 | 20 ℃/HFD F1 | 162.24±10.10 | 0.683 | 30 |
|  |  |  |  |  |  |
|  | EXP.2 | 20 ℃/Control P0 | 162.62±7.62 |  | 30 |
|  | EXP.2 | 20 ℃/HFD P0 | 371.85±14.91 | <0.0001 | 31 |
|  | EXP.2 | 20 ℃/Control F1 | 163.50±9.96 |  | 30 |
|  | EXP.2 | 20 ℃/HFD F1 | 163.22±8.94 | 0.790 | 31 |
|  |  |  |  |  |  |
|  | EXP.3 | 20 ℃/Control P0 | 160.76±9.38 |  | 30 |
|  | EXP.3 | 20 ℃/HFD P0 | 367.85±14.99 | <0.0001 | 30 |
|  | EXP.3 | 20 ℃/Control F1 | 163.18±8.09 |  | 30 |
|  | EXP.3 | 20 ℃/HFD F1 | 160.64±9.86 | 0.281 | 30 |
|  |  |  |  |  |  |
| Supplementary  Fig. 3c | ***daf-16* (OE); *sbp-1 RNAi*** | |  |  |  |
|  | EXP.1 | 20 ℃/Control P0 | 54.90±13.61 |  | 31 |
|  | EXP.1 | 20 ℃/HFD P0 | 158.47±38.26 | <0.0001 | 36 |
|  | EXP.1 | 20 ℃/Control F1 | 53.18±11.19 |  | 30 |
|  | EXP.1 | 20 ℃/HFD F1 | 47.65±5.27 | 0.016 | 31 |
|  |  |  |  |  |  |
|  |  |  |  |  |  |
|  | EXP.2 | 20 ℃/Control P0 | 59.27±9.00 |  | 33 |
|  | EXP.2 | 20 ℃/HFD P0 | 162.76±33.87 | <0.0001 | 33 |
|  | EXP.2 | 20 ℃/Control F1 | 49.02±8.94 |  | 30 |
|  | EXP.2 | 20 ℃/HFD F1 | 50.81±6.40 | 0.377 | 30 |
|  |  |  |  |  |  |
|  | EXP.3 | 20 ℃/Control P0 | 62.32±15.44 |  | 32 |
|  | EXP.3 | 20 ℃/HFD P0 | 161.85±35.17 | <0.0001 | 34 |
|  | EXP.3 | 20 ℃/Control F1 | 52.36±6.19 |  | 30 |
|  | EXP.3 | 20 ℃/HFD F1 | 51.32±5.79 | 0.566 | 31 |
|  |  |  |  |  |  |
|  | **N2** |  |  |  |  |
|  | **P0 (OP50)→F1 (corresponding RNAi bacteria)** | | |  |  |
| Fig 2f & Supplementary Fig. 4b | **HT115** | |  |  |  |
|  | EXP.1 | 20 ℃/Control F1 | 57.03±13.73 |  | 30 |
|  | EXP.1 | 20 ℃/HFD F1 | 112.27±25.93 | <0.0001 | 30 |
|  | EXP.2 | 20 ℃/Control F1 | 61.23±13.19 |  | 31 |
|  | EXP.2 | 20 ℃/HFD F1 | 112.39±21.27 | <0.0001 | 30 |
|  | EXP.3 | 20 ℃/Control F1 | 57.83±16.04 |  | 32 |
|  | EXP.3 | 20 ℃/HFD F1 | 114.37±21.423 | <0.0001 | 31 |
|  |  | |  |  |  |
|  | ***daf-16* RNAi** | |  |  |  |
| Fig. 2f | EXP.1 | 20 ℃/Control F1 | 57.24±16.63 |  | 31 |
|  | EXP.1 | 20 ℃/HFD F1 | 54.47±21.49 | 0.107 | 31 |
|  | EXP.2 | 20 ℃/Control F1 | 69.46±14.10 |  | 30 |
|  | EXP.2 | 20 ℃/HFD F1 | 65.66±18.41 | 0.205 | 30 |
|  | EXP.3 | 20 ℃/Control F1 | 55.98±20.16 |  | 31 |
|  | EXP.3 | 20 ℃/HFD F1 | 59.98±18.69 | 0.419 | 31 |
|  |  |  |  |  |  |
| Supplementary  Fig. 7a | ***wdr-5.1* RNAi** | |  |  |  |
|  | EXP.1 | 20 ℃/Control F1 | 101.35±24.12 |  | 29 |
|  | EXP.1 | 20 ℃/HFD F1 | 99.76±40.52 | 0.202 | 29 |
|  | EXP.2 | 20 ℃/Control F1 | 119.69±35.20 |  | 36 |
|  | EXP.2 | 20 ℃/HFD F1 | 123.38±29.30 | 0.646 | 36 |
|  | EXP.3 | 20 ℃/Control F1 | 112.48±35.03 |  | 35 |
|  | EXP.3 | 20 ℃/HFD F1 | 118.09±42.19 | 0.850 | 34 |
|  |  |  |  |  |  |
| Supplementary  Fig. 4c | ***nhr-49* RNAi** | |  |  |  |
|  | EXP.1 | 20 ℃/Control F1 | 102.43±20.03 |  | 33 |
|  | EXP.1 | 20 ℃/HFD F1 | 110.63±37.53 | 0.345 | 31 |
|  | EXP.2 | 20 ℃/Control F1 | 102.82±19.01 |  | 34 |
|  | EXP.2 | 20 ℃/HFD F1 | 108.10±27.32 | 0.414 | 31 |
|  | EXP.3 | 20 ℃/Control F1 | 104.35±26.95 |  | 33 |
|  | EXP.3 | 20 ℃/HFD F1 | 113.34±26.81 | 0.809 | 34 |
|  |  |  |  |  |  |
| Supplementary  Fig. 4d | ***nhr-80* RNAi** | |  |  |  |
|  | EXP.1 | 20 ℃/Control F1 | 103.18±27.62 |  | 31 |
|  | EXP.1 | 20 ℃/HFD F1 | 99.51±28.53 | 0.852 | 31 |
|  | EXP.2 | 20 ℃/Control F1 | 106.07±24.16 |  | 36 |
|  | EXP.2 | 20 ℃/HFD F1 | 105.62±29.74 | 0.402 | 36 |
|  | EXP.3 | 20 ℃/Control F1 | 101.76±21.25 |  | 36 |
|  | EXP.3 | 20 ℃/HFD F1 | 108.49±27.03 | 0.305 | 37 |
| Supplementary  Fig. 4e | ***sbp-1* RNAi** | |  |  |  |
|  | EXP.1 | 20 ℃/Control F1 | 38.68±12.55 |  | 42 |
|  | EXP.1 | 20 ℃/HFD F1 | 33.99±11.15 | 0.518 | 44 |
|  | EXP.2 | 20 ℃/Control F1 | 37.98±12.74 |  | 36 |
|  | EXP.2 | 20 ℃/HFD F1 | 32.39±9.11 | 0.052 | 40 |
|  | EXP.3 | 20 ℃/Control F1 | 39.85±11.80 |  | 37 |
|  | EXP.3 | 20 ℃/HFD F1 | 36.75±7.13 | 0.107 | 37 |
|  |  |  |  |  |  |
|  | ***fat-5* RNAi** | |  |  |  |
| Fig. 3g | EXP.1 | 20 ℃/Control F1 | 102.22±21.62 |  | 34 |
|  | EXP.1 | 20 ℃/HFD F1 | 97.28±21.86 | 0.670 | 31 |
|  | EXP.2 | 20 ℃/Control F1 | 95.52±22.40 |  | 34 |
|  | EXP.2 | 20 ℃/HFD F1 | 101.36±12.72 | 0.189 | 32 |
|  | EXP.3 | 20 ℃/Control F1 | 98.21±26.17 |  | 37 |
|  | EXP.3 | 20 ℃/HFD F1 | 97.71±17.07 | 0.941 | 33 |
|  |  |  |  |  |  |
|  | **N2** |  |  |  |  |
|  | **P0 (corresponding RNAi bacteria)→F1 (OP50)** | | | |  |
|  | **HT115** |  |  |  |  |
|  | EXP.1 | 20 ℃/Control P0 | 63.58±17.54 |  | 34 |
|  | EXP.1 | 20 ℃/HFD P0 | 252.59±80.61 | <0.0001 | 37 |
|  | EXP.1 | 20 ℃/Control F1 | 109.68±28.44 |  | 29 |
|  | EXP.1 | 20 ℃/HFD F1 | 208.61±73.08 | <0.0001 | 29 |
|  | EXP.2 | 20 ℃/Control P0 | 61.18±17.84 |  | 32 |
|  | EXP.2 | 20 ℃/HFDP0 | 253.86±82.56 | <0.0001 | 33 |
|  | EXP.2 | 20 ℃/Control F1 | 113.17±30.76 |  | 31 |
|  | EXP.2 | 20 ℃/HFD F1 | 200.12±72.55 | <0.0001 | 30 |
|  |  |  |  |  |  |
|  | ***daf-16* RNAi** | |  |  |  |
| Fig. 2k | EXP.1 | 20 ℃/Control P0 | 58.12±16.15 |  | 30 |
|  | EXP.1 | 20 ℃/HFD P0 | 54.32±21.84 | 0.285 | 30 |
|  | EXP.1 | 20 ℃/Control F1 | 96.13±16.98 |  | 30 |
|  | EXP.1 | 20 ℃/HFD F1 | 93.25±19.93 | 0.669 | 30 |
|  | EXP.2 | 20 ℃/Control P0 | 59.54±13.93 |  | 28 |
|  | EXP.2 | 20 ℃/HFDP0 | 57.62±11.12 | 0.649 | 27 |
|  | EXP.2 | 20 ℃/Control F1 | 92.57±15.49 |  | 34 |
|  | EXP.2 | 20 ℃/HFD F1 | 91.59±20.89 | 0.679 | 34 |
|  | EXP.3 | 20 ℃/Control P0 | 56.97±13.74 |  | 35 |
|  | EXP.3 | 20 ℃/HFDP0 | 55.15±17.42 | 0.986 | 38 |
|  | EXP.3 | 20 ℃/Control F1 | 94.88±15.89 |  | 36 |
|  | EXP.3 | 20 ℃/HFD F1 | 96.91±19.51 | 0.838 | 36 |
|  |  | |  |  |  |
|  | ***wdr-5.1* RNAi** | |  |  |  |
| Supplementary  Fig. 7b | EXP.1 | 20 ℃/Control P0 | 99.06±20.29 |  | 32 |
|  | EXP.1 | 20 ℃/HFDP0 | 235.94±59.62 | <0.0001 | 37 |
|  | EXP.1 | 20 ℃/Control F1 | 103.09±25.90 |  | 31 |
|  | EXP.1 | 20 ℃/HFD F1 | 105.02±21.73 | 0.682 | 31 |
|  | EXP.2 | 20 ℃/Control P0 | 123.83±36.66 |  | 34 |
|  | EXP.2 | 20 ℃/HFDP0 | 222.41±52.06 | <0.0001 | 31 |
|  | EXP.2 | 20 ℃/Control F1 | 101.75±22.41 |  | 37 |
|  | EXP.2 | 20 ℃/HFD F1 | 106.05±22.45 | 0.527 | 37 |
|  | EXP.3 | 20 ℃/Control P0 | 112.95±37.12 |  | 32 |
|  | EXP.3 | 20 ℃/HFDP0 | 216.90±53.20 | <0.0001 | 32 |
|  | EXP.3 | 20 ℃/Control F1 | 102.58±22.20 |  | 35 |
|  | EXP.3 | 20 ℃/HFD F1 | 111.55±23.08 | 0.071 | 36 |
|  |  |  |  |  |  |
|  | ***nhr-49* RNAi** | |  |  |  |
| Fig. 2j | EXP.1 | 20 ℃/Control P0 | 103.89±18.90 |  | 31 |
|  | EXP.1 | 20 ℃/HFDP0 | 205.18±57.96 | <0.0001 | 31 |
|  | EXP.1 | 20 ℃/Control F1 | 112.89±22.24 |  | 31 |
|  | EXP.1 | 20 ℃/HFD F1 | 153.47±39.85 | 0.001 | 31 |
|  | EXP.2 | 20 ℃/Control P0 | 97.28±25.65 |  | 31 |
|  | EXP.2 | 20 ℃/HFDP0 | 190.20±71.00 | <0.0001 | 31 |
|  | EXP.2 | 20 ℃/Control F1 | 115.05±33.01 |  | 31 |
|  | EXP.2 | 20 ℃/HFD F1 | 160.27±37.92 | 0.002 | 31 |
|  | EXP.3 | 20 ℃/Control P0 | 111.90±25.58 |  | 34 |
|  | EXP.3 | 20 ℃/HFDP0 | 197.87±55.19 | <0.0001 | 31 |
|  | EXP.3 | 20 ℃/Control F1 | 130.58±25.66 |  | 33 |
|  | EXP.3 | 20 ℃/HFD F1 | 168.57±35.59 | 0.0156 | 32 |
|  |  |  |  |  |  |
|  | ***nhr-80* RNAi** | |  |  |  |
| Fig. 2i | EXP.1 | 20 ℃/Control P0 | 106.11±26.59 |  | 31 |
|  | EXP.1 | 20 ℃/HFDP0 | 271.06±65.48 | <0.0001 | 31 |
|  | EXP.1 | 20 ℃/Control F1 | 117.19±23.39 |  | 36 |
|  | EXP.1 | 20 ℃/HFD F1 | 184.76±41.94 | <0.0001 | 36 |
|  | EXP.2 | 20 ℃/Control P0 | 104.05±23.64 |  | 37 |
|  | EXP.2 | 20 ℃/HFD P0 | 288.38±66.01 | <0.0001 | 36 |
|  | EXP.2 | 20 ℃/Control F1 | 120.13±26.63 |  | 31 |
|  | EXP.2 | 20 ℃/HFD F1 | 169.25±53.84 | 0.0212 | 30 |
|  | EXP.3 | 20 ℃/Control P0 | 103.55±27.54 |  | 35 |
|  | EXP.3 | 20 ℃/HFDP0 | 277.53±75.46 | <0.0001 | 34 |
|  | EXP.3 | 20 ℃/Control F1 | 115.34±18.26 |  | 34 |
|  | EXP.3 | 20 ℃/HFD F1 | 179.53±45.32 | <0.0001 | 34 |
|  |  | |  |  |  |
|  | ***sbp-1* RNAi** | |  |  |  |
| Fig. 2h | EXP.1 | 20 ℃/Control P0 | 41.58±12.05 |  | 36 |
|  | EXP.1 | 20 ℃/HFDP0 | 120.97±34.50 | <0.0001 | 31 |
|  | EXP.1 | 20 ℃/Control F1 | 92.24±22.16 |  | 33 |
|  | EXP.1 | 20 ℃/HFD F1 | 93.96±24.34 | 0.478 | 31 |
|  | EXP.2 | 20 ℃/Control P0 | 42.31±12.29 |  | 35 |
|  | EXP.2 | 20 ℃/HFDP0 | 112.25±35.79 | <0.0001 | 33 |
|  | EXP.2 | 20 ℃/Control F1 | 91.20±22.08 |  | 35 |
|  | EXP.2 | 20 ℃/HFD F1 | 98.46±26.85 | 0.0759 | 33 |
|  | EXP.3 | 20 ℃/Control P0 | 41.36±11.53 |  | 36 |
|  | EXP.3 | 20 ℃/HFDP0 | 118.75±43.06 | <0.0001 | 35 |
|  | EXP.3 | 20 ℃/Control F1 | 94.82±23.23 |  | 39 |
|  | EXP.3 | 20 ℃/HFD F1 | 100.75±29.00 | 0.708 | 39 |
|  |  |  |  |  |  |
|  | ***fat-5* RNAi** | |  |  |  |
| Fig. 3h | EXP.1 | 20 ℃/Control P0 | 105.92±21.25 |  | 31 |
|  | EXP.1 | 20 ℃/HFDP0 | 232.93±63.12 | <0.0001 | 31 |
|  | EXP.1 | 20 ℃/Control F1 | 99.37±22.13 |  | 31 |
|  | EXP.1 | 20 ℃/HFD F1 | 164.42±39.50 | <0.0001 | 31 |
|  | EXP.2 | 20 ℃/Control P0 | 94.70±23.94 |  | 32 |
|  | EXP.2 | 20 ℃/HFDP0 | 190.41±33.71 | <0.0001 | 32 |
|  | EXP.2 | 20 ℃/Control F1 | 96.28±19.31 |  | 32 |
|  | EXP.2 | 20 ℃/HFD F1 | 167.37±35.85 | <0.0001 | 32 |
|  | EXP.3 | 20 ℃/Control P0 | 95.11±23.45 |  | 36 |
|  | EXP.3 | 20 ℃/HFDP0 | 242.37±64.85 | <0.0001 | 36 |
|  | EXP.3 | 20 ℃/Control F1 | 105.71±20.20 |  | 31 |
|  | EXP.3 | 20 ℃/HFD F1 | 157.73±37.35 | <0.0001 | 31 |
|  |  |  |  |  |  |
|  |  |  |  |  |  |
|  | ***fat-6* RNAi** | |  |  |  |
| Supplementary  Fig. 4g | EXP.1 | 20 ℃/Control P0 | 49.23±10.17 |  | 32 |
|  | EXP.1 | 20 ℃/HFDP0 | 191.17±39.58 | <0.0001 | 31 |
|  | EXP.1 | 20 ℃/Control F1 | 88.98±17.57 |  | 32 |
|  | EXP.1 | 20 ℃/HFD F1 | 152.60±57.12 | <0.0001 | 31 |
|  | EXP.2 | 20 ℃/Control P0 | 51.31±12.99 |  | 34 |
|  | EXP.2 | 20 ℃/HFDP0 | 198.34±43.17 | <0.0001 | 33 |
|  | EXP.2 | 20 ℃/Control F1 | 93.48±19.60 |  | 33 |
|  | EXP.2 | 20 ℃/HFD F1 | 172.97±60.06 | <0.0001 | 33 |
|  |  |  |  |  |  |
|  | ***fat-7* RNAi** | |  |  |  |
| Supplementary  Fig. 4h | EXP.1 | 20 ℃/Control P0 | 52.02±11.76 |  | 31 |
|  | EXP.1 | 20 ℃/HFDP0 | 182.62±46.67 | <0.0001 | 31 |
|  | EXP.1 | 20 ℃/Control F1 | 96.27±24.76 |  | 31 |
|  | EXP.1 | 20 ℃/HFD F1 | 150.04±47.36 | <0.0001 | 31 |
|  | EXP.2 | 20 ℃/Control P0 | 51.45±11.42 |  | 34 |
|  | EXP.2 | 20 ℃/HFDP0 | 181.57±46.07 | <0.0001 | 33 |
|  | EXP.2 | 20 ℃/Control F1 | 94.75±23.68 |  | 34 |
|  | EXP.2 | 20 ℃/HFD F1 | 147.86±40.70 | <0.0001 | 34 |
