# Supplementary material for: Histone H3K4me3 modification is a transgenerational epigenetic signal for lipid metabolism in Caenorhabditis elegans
Source: Nat Commun. 2022 Feb 9;13:768. doi: 10.1038/s41467-022-28469-4 (PMC8828817; doi:10.1038/s41467-022-28469-4)
Supplement: Supplementary file 4 — Source Data [file 41467_2022_28469_MOESM4_ESM.zip › source data/Uncropped western blot figure.docx]

**Uncropped western blot figure**

**
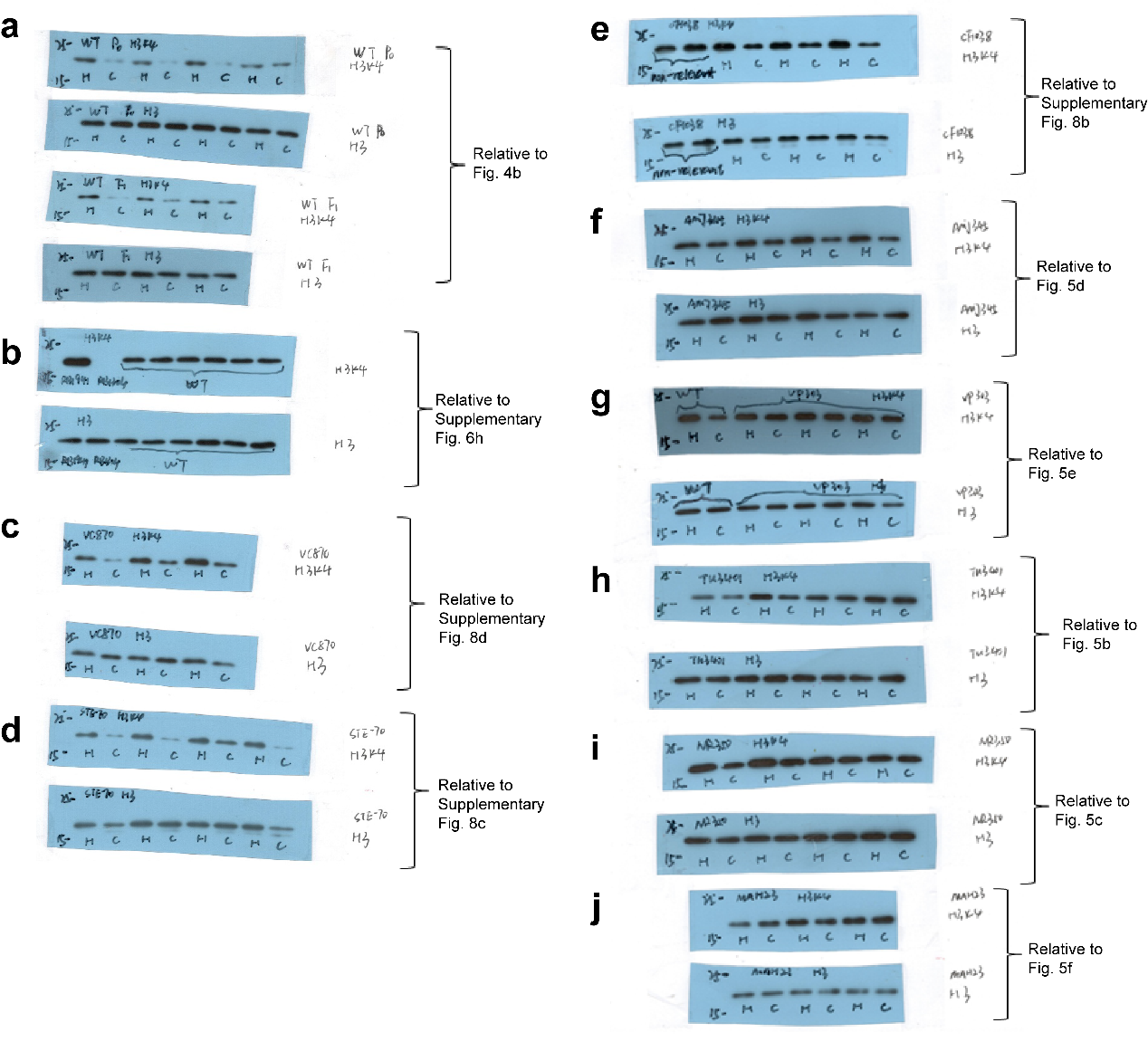
**

**
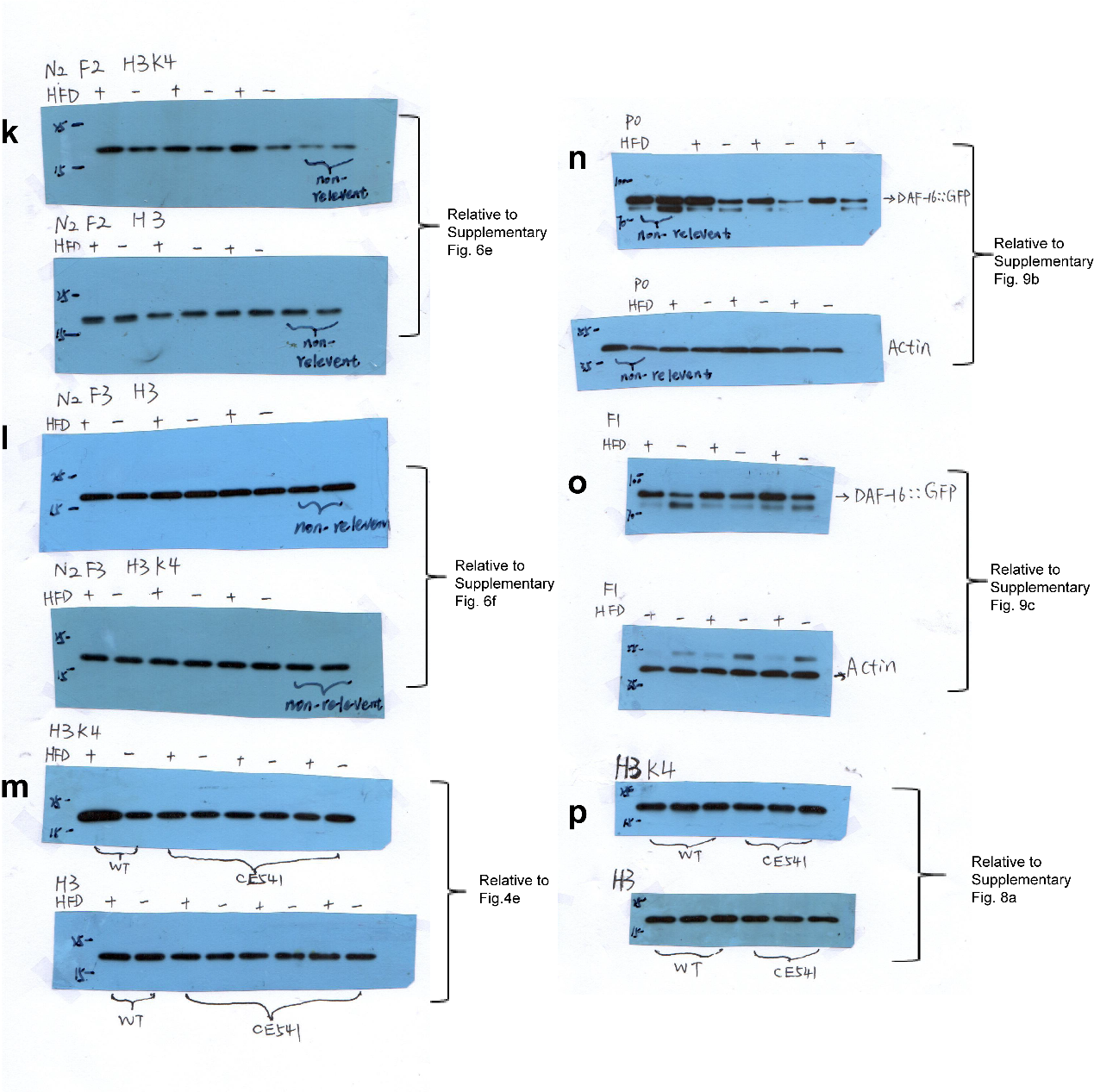
**

**Uncropped western blot figure. (a)** The level of histone H3K4me3 modification in WT animals fed with or without the HFD, relative to Fig. 4b. **(b)** The level of histone H3K4me3 modification in *rbr-2* mutant, *wdr-5.1* mutant and WT N2 fed with normal OP50, relative to Supplementary Fig. 4h. **(c)** The level of histone H3K4me3 modification in *nhr-49* mutant fed with or without the HFD, relative to supplementary Fig. 6d. **(d)** The level of histone H3K4me3 modification in *nhr-80* mutant fed with or without the HFD, relative to supplementary Fig. 6c. **(e)** The level of histone H3K4me3 modification in *daf-16* mutant fed with or without the HFD, relative to supplementary Fig. 6b. **(f)** The level of histone H3K4me3 modification in AMJ345 strain fed with or without the HFD, relative to Fig. 5d. **(g)** The level of histone H3K4me3 modification in VP303 strain fed with or without the HFD, relative to Fig. 5e. **(h)** The level of histone H3K4me3 modification in TU3401 strain fed with or without the HFD, relative to Fig. 5b. **(i)** The level of histone H3K4me3 modification in NR350 strain fed with or without the HFD, relative to Fig. 5c. **(j)** The level of histone H3K4me3 modification in MAH23 strain fed with or without the HFD, relative to Fig. 5f. **(k, l)** The level of histone H3K4me3 modification in WT animals fed with or without the HFD, relative to Supplementary Fig. 6e and 6f, respectively. **(m)** The level of histone H3K4me3 modification in *sbp-1*mutant and WT N2 fed with or without the HFD, relative to Fig. 4e. **(n, o)** The level of DAF-16::GFP in TJ356 transgenetic animals fed with or without the HFD, relative to Supplementary Fig. 9b and 9c, respectively. **(p)** The level of histone H3K4me3 modification in *sbp-1*mutant and WT N2 fed with normal OP50, relative to Supplementary Fig. 8a.
